# Histone H2A Mono-Ubiquitination Is a Crucial Step to Mediate PRC1-Dependent Repression of Developmental Genes to Maintain ES Cell Identity

**DOI:** 10.1371/journal.pgen.1002774

**Published:** 2012-07-26

**Authors:** Mitsuhiro Endoh, Takaho A. Endo, Tamie Endoh, Kyo-ichi Isono, Jafar Sharif, Osamu Ohara, Tetsuro Toyoda, Takashi Ito, Ragnhild Eskeland, Wendy A. Bickmore, Miguel Vidal, Bradley E. Bernstein, Haruhiko Koseki

**Affiliations:** 1 Laboratory for Developmental Genetics, RIKEN Research Center for Allergy and Immunology, Yokohama, Japan; 2 Core Research for Evolutional Science and Technology, Japan Science and Technology Agency, Yokohama, Japan; 3 RIKEN Bioinformatics and System Engineering Division, Yokohama, Japan; 4 Laboratories for Immunogenomics, RIKEN Research Center for Allergy and Immunology, Yokohama, Japan; 5 Department of Biochemistry, Nagasaki University School of Medicine, Nagasaki, Japan; 6 MRC Human Genetics Unit, Institute of Genetics and Molecular Medicine, University of Edinburgh, Edinburgh, United Kingdom; 7 Cell Proliferation and Development, Centro de Investigaciones Biologicas, Consejo Superior de Investigaciones Científicas, Madrid, Spain; 8 Research Unit for Immunoepigenetics, RIKEN Research Center for Allergy and Immunology, Yokohama, Japan; 9 Molecular Pathology Unit and Center for Cancer Research, Massachusetts General Hospital, Charlestown, Massachusetts, United States of America; 10 Department of Pathology, Harvard Medical School, Boston, Massachusetts, United States of America; 11 Broad Institute of Harvard and MIT, Cambridge, Massachusetts, United States of America; Friedrich Miescher Institute for Biomedical Research, Switzerland

## Abstract

Two distinct Polycomb complexes, PRC1 and PRC2, collaborate to maintain epigenetic repression of key developmental loci in embryonic stem cells (ESCs). PRC1 and PRC2 have histone modifying activities, catalyzing mono-ubiquitination of histone H2A (H2AK119u1) and trimethylation of H3 lysine 27 (H3K27me3), respectively. Compared to H3K27me3, localization and the role of H2AK119u1 are not fully understood in ESCs. Here we present genome-wide H2AK119u1 maps in ESCs and identify a group of genes at which H2AK119u1 is deposited in a Ring1-dependent manner. These genes are a distinctive subset of genes with H3K27me3 enrichment and are the central targets of Polycomb silencing that are required to maintain ESC identity. We further show that the H2A ubiquitination activity of PRC1 is dispensable for its target binding and its activity to compact chromatin at *Hox* loci, but is indispensable for efficient repression of target genes and thereby ESC maintenance. These data demonstrate that multiple effector mechanisms including H2A ubiquitination and chromatin compaction combine to mediate PRC1-dependent repression of genes that are crucial for the maintenance of ESC identity. Utilization of these diverse effector mechanisms might provide a means to maintain a repressive state that is robust yet highly responsive to developmental cues during ES cell self-renewal and differentiation.

## Author Summary

Polycomb-group (PcG) proteins play essential roles in the epigenetic regulation of gene expression during development. PcG proteins form two distinct multimeric complexes, PRC1 and PRC2. In the widely accepted hierarchical model, PRC2 is recruited to specific genomic locations and catalyzes trimethylation of H3 lysine 27 (H3K27me3), thereby creating binding sites for PRC1, which then catalyzes mono-ubiquitination of histone H2A (H2AK119u1). Recently, PRC1 has been shown to be able to compact chromatin structure at target loci independently of its histone ubiquitination activity. Therefore, the role of H2AK119u1 still remains unclear. To gain insight into this issue, we used ChIP-on-chip analysis to map H2AK119u1 genome-wide in mouse ES cells (ESCs). The data demonstrate that H2AK119u1 occupies a distinctive subset of genes with H3K27me3 enrichment. These genes are the central targets of Polycomb silencing to maintain ESC identity. We further show that the H2A ubiquitination activity of PRC1 is dispensable for its target binding and its activity to compact chromatin at *Hox* loci, but is indispensable for efficient repression of target genes and therefore ESC maintenance. We propose that multiple effector mechanisms including H2A ubiquitination and chromatin compaction combine to mediate PRC1-dependent repression of developmental genes to maintain the identity of ESCs.

## Introduction

Embryonic stem cells (ESCs) can undergo unlimited self-renewal while maintaining their pluripotent and undifferentiated states, features that are consistent with their origin within the inner cell mass of the blastocyst. Increasing evidence suggests that in addition to the core gene regulatory circuitry composed of Oct3/4, Sox2, Nanog and other transcription factors, Polycomb group proteins critically contribute to maintain the undifferentiated state of ESCs by silencing genes that are involved in development and/or transcription [1], [2], [3], [4], [5], [6]. Polycomb-mediated repression of these genes is also essential to preserve the ability of ES cells to differentiate in response to extracellular cues [7], [8], [9].

Polycomb group proteins are chromatin-modifiers that mediate transcriptional repression. They form at least two types of multimeric complexes, the Polycomb repressive complexes-1 (PRC1) and -2 (PRC2), the core components of which are conserved from *Drosophila* to human [10], [11], [12], [13], [14]. PRC2 contains Ezh2 or -1, which catalyze trimethylation of histone H3 lysine 27 (H3K27me3), a posttranslational modification that is thought to be recognized by the chromo-domain (CHD) protein components of PRC1 [12], [13], [14], [15], [16]. Within PRC1, Ring1B and –A act as major E3 ubiquitin ligases for histone H2A mono-ubiquitination at lysine 119 (H2AK119u1) [17], [18]. Conditional depletion of Ring1B and –A in ESCs leads to global loss of H2AK119u1 and concurrent derepression of ‘bivalent’ genes enriched for both H3K27me3 and H3K4me3 [5], [19]. H2AK119u1 deposition has been shown to localize to the inactive X chromosome (Xi), the XY body, and several silenced ‘bivalent’ loci in mouse ESCs [19], [20], [21]. Recent genome-wide H2AK119u1 analysis in MEFs (mouse embryonic fibroblast) has revealed Bmi1-dependent deposition of H2AK119u1 at the promoter regions of many repressed genes [22]. These findings suggest that H2AK119u1 could be a part of the regulatory process that is required for PRC1-mediated repression.

However, the role of H2AK119u1 in PRC1-mediated repression is still controversial. A recent study has reported that Ring1B can compact chromatin structure of the *Hox* loci and repress *Hox* expression independent of its E3 activity [23]. This idea has been supported by a previous study which showed that PRC1 components can compress nucleosomal templates assembled from tail-less histones into a form that is refractory to chromatin remodeling *in vitro* [24]. This hypothesis, however, needs rigorous validation because this study was performed by using *Ring1B* single knockout (KO) cells, in which Ring1A-catalyzed H2AK119u1 still remained in a lower level [5], [17], [20], [25]. In this experimental setup, Ring1A and associated H2AK119u1 may potentially complement Ring1B-mediated chromatin compaction of *Hox* genes to mediate their repression. Consistently, ESCs are capable to retain ESC-like morphology and LIF-dependent proliferation upon depletion of Ring1B but not doubly depletion of Ring1B and –A [5], [9], [20], [26]. Therefore, to properly estimate in which extent H2AK119u1 contributes to PRC1-dependent repression in ESCs, and experimental platform that excludes Ring1A is necessary.

In this study, we first determined the localization of H2AK119u1 in ESCs by ChIP-on-chip analysis and found the H2AK119u1-bound genes as core targets of PRC1-dependent repression. We further demonstrated that catalytic activity of PRC1 towards H2A is essential for silencing of target loci and maintenance of ESCs. We also found PRC1-mediated H2AK119u1 is complemented by independent functions of PRC1 that contribute to gene silencing and chromatin compaction, most notably at *Hox* loci. We propose that PRC1 combines diverse effecter mechanisms to mediate robust repression of target genes and stable maintenance of undifferentiated status of ESCs.

## Results

### Ring1-mediated H2AK119u1 demarcates central targets of PRC1 in ESCs

Global H2AK119u1 distribution has been reported only for MEFs and the human teratocarcinoma NT2 cell line [22], [27], but not for mouse ESCs. We, therefore, used ChIP-on-chip analysis to clarify H2AK119u1 deposition around transcription start sites (TSS) in mouse ESCs by using an Agilent mouse promoter array and an E6C5 monoclonal antibody (mAb) or a rabbit polyclonal antibody [28]. *Ring1A/B*-dKO ESCs, in which H2AK119u1 is apparently undetectable, were used as a negative control [5], [19]. *Ring1A/B-*dKO ESCs were induced by treating *Ring1A^−/−^;Ring1B^fl/fl^;Rosa26::CreERT2* ESCs with 4-hydroxy-tamoxifen (OHT), which rapidly activates CreERT2 and catalyzes loxP recombination at *Ring1B* locus [5]. Distribution of Ring1B, H3K27me3 and H2A were re-examined to obtain a reference data set.

**Figure 1:**
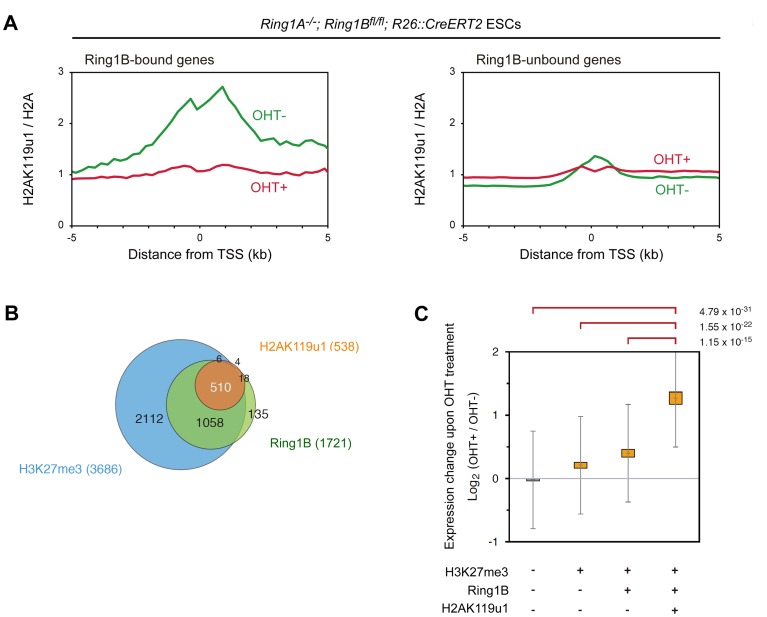
Global mapping of Ring1B-dependent H2AK119u1 deposition in ESCs reveals that genes occupied by H2AK119u1 represent central targets of PRC1. (A) ChIP-on-chip analysis showing the average of H2AK119u1 distributions at the promoter regions (from −5 kb to +5 kb relative to TSS) of Ring1B-bound and –unbound genes in *Ring1A^−/−^* (OHT−: green line) and *Ring1A/B*-dKO (OHT+: red line) ESCs. Enrichment of H2AK119u1 (obtained by E6C5 mAb) and H2A is expressed relative to input DNA, and H2AK119u1 is normalized to H2A. (B) Venn diagram representing the overlap among genes occupied by Ring1B, H2AK119u1 and H3K27me3. Numbers in parentheses represent the total number of genes occupied by each one. (C) Graphic representation of expression changes induced by *Ring1B* depletion (2 days after OHT treatment) for each subset of genes classified by the presence (+) or absence (−) of Ring1B, H2AK119u1 and H3K27me3 is shown. The average, deviation and distribution of the expression changes for the respective subsets of genes determined by microarray analysis are shown. The 95% Confidence interval (CI) and standard deviation (SD) for the average value of the expression change are indicated. Significant (*P*<0.001) and insignificant (*P*≥0.01) expression changes were determined by the Student's *t*-test and are indicated in orange and grey, respectively. *P*-values for the difference of expression changes between the indicated 2 groups are calculated by the Student's *t*-test and are indicated above each graph.

E6C5-ChIP signals at the promoter regions of known target loci, *Hoxa9*, *Pax9* and *Tbx3* show that H2AK119u1 deposition is readily detectable in *Ring1A^−/−^* ESCs but not in *Ring1A/B*-dKO (dKO) ESCs (Figure S1). These results were validated by ChIP-qPCR as shown in Figure S2A. We calculated the averages of E6C5-ChIP signals in Ring1B-bound and –unbound genes and found higher H2AK119u1 deposition in Ring1B-bound genes in *Ring1A^−/−^* than in *Ring1A/B*-dKO (dKO) whereas little difference was noted for unbound genes (Figure 1A; Figure S3A and S3B). These data therefore appear to reflect H2AK119u1 deposition that depends on Ring1B. For detailed investigation of the genes that exhibit H2AK119u1 enrichment, we examined the gene-wise distribution of E6C5-ChIP signals after subtraction of the background enrichment value and identified 538 target genes (Figure 1B; Figure S3C; the list of genes is shown in Table S1). The results with the E6C5 mAb were re-confirmed by using a rabbit antiserum that recognizes H2AK119u1 [28]. With this reagent, 524 genes were found to be bound by H2AK119u1, and these genes significantly overlapped with those identified by the E6C5 mAb (Figure S3D; Table S1). Taken together, using the above methods we determined a set of genes in ESCs that have H2AK119u1 deposition around their TSS.

We went on to examine the correlation of genes enriched for H2AK119u1 (H2AK119u1+) with those having Ring1B (Ring1B+) and H3K27me3 (H3K27me3+) depositions. We found that genes bound by Ring1B and H3K27me3 identified in this study were significantly overlapped with those reported in previous studies (Figure S3E, F). We identified 1721 and 3686 genes bound by Ring1B and H3K27me3, respectively, and found H2AK119u1+ genes as a subset of the Ring1B+ genes (Figure 1B; Figure S3C; Table S1). Since most Ring1B+ genes define a subset of H3K27me3+ genes, H3K27me3+ genes could be subdivided into three distinct layers, H2AK119u1+Ring1B+H3K27me3+ (Triple positive; TP), H2AK119u1-Ring1B+H3K27me3+ (Double positive; DP) and H2AK119u1-Ring1B-H3K27me3+ (Single positive; SP). We finally confirmed the quantitative difference of H2AK119u1 level at TP genes against DP or SP genes by ChIP-qPCR analysis at selected genes (Figure S2A). Although we cannot exclude a possibility that we failed to detect a low level of H2AK119u1 at some DP genes, our data demonstrate that H3K27me3+ gene promoters are not uniformly occupied by Ring1B and H2AK119u1.

We investigated functional properties of H2AK119u1+ genes among Polycomb targets. Scattered plot analysis for gene-wise deposition of H3K27me3 and Ring1B revealed that H2AK119u1 targets were significantly enriched among genes that have high levels of both Ring1B and H3K27me3 occupancy (Figure S4). This suggests that TP genes represent the central targets for Polycomb repression. We compared the impact of PRC1 loss among these subsets by examining the gene expression profiles in *Ring1A/B*-dKO ESCs (Figure 1C; Figure S2B). We found significant de-repression (p<0.001) of TP, DP and SP genes but no significant changes in H3K27me3-negative genes. It is worth noting that the degree of de-repression of the TP genes was significantly higher than that of the DP and SP genes (Figure 1C). Gene ontology (GO) based analyses confirmed that TP genes are most significantly enriched for functions in transcription and/or development (Figure S5). Of note, *Cdx2* and *Gata6*, which are known to be repressed by Oct3/4 and Nanog [29], [30], are occupied by H2AK119u1, suggesting that H2AK119u1 might be involved in maintaining ESC properties by suppressing differentiation of ESCs.

### E3 activity of Ring1B and H2AK119u1 are dispensable for PRC1 target binding

**Figure 2:**
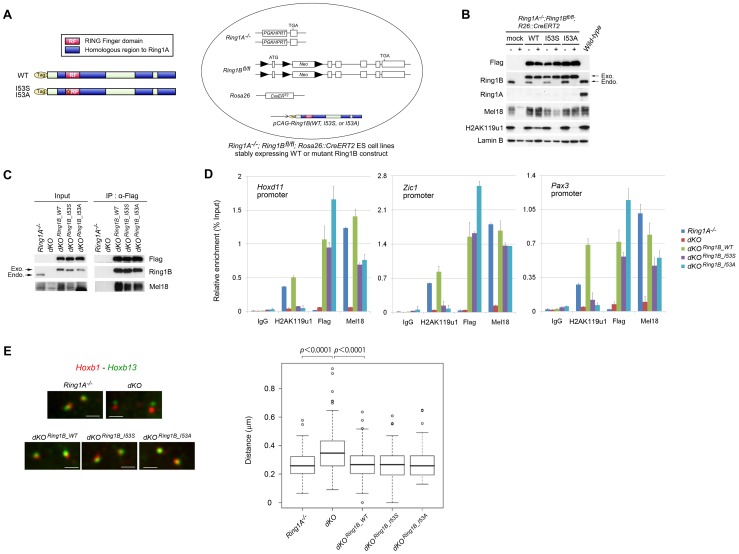
Generation of ESCs expressing catalytically inactive Ring1B. (A) Schematic representation of 3xFlag-tagged Ring1B, showing wild-type and point-mutant derivatives. Each of these construct was stably transfected into *Ring1A^−/−^; Ring1B^fl/fl^; R26::CreERT2* ESCs. (B) Immunoblot analysis of Ring1A, Ring1B, Flag, H2AK119u1 and Lamin B protein levels in whole cell lysates of *wild-type* and *Ring1A^−/−^; Ring1B^fl/fl^; R26::CreERT2* ESC lines expressing mock, WT, I53S, or I53A Ring1B with or without OHT treatment (OHT+ and −, respectively). (C) Immunoprecipitation (IP) analysis showing the association of exogenous Ring1B WT, I53S or I53A with an endogenous PRC1 component Mel18. Extracts of OHT-untreated (−) and -treated [(+); day 2] *Ring1A^−/−^; Ring1B^fl/fl^; R26::CreERT2* ESC lines expressing each of the constructs were immunoprecipitated with anti-Flag antibody. Resulting precipitates (IP) and lysates (Input) were immunoblotted with antibodies against Flag, Ring1B and Mel18. (D) Association of Flag-tagged proteins in *Ring1A^−/−^; Ring1B^fl/fl^; R26::CreERT2* ESC lines stably expressing mock, Flag-tagged Ring1B WT, I53S, or I53A with promoter regions of their representative target genes before (−) or after (+) OHT treatment (day 2) as determined by ChIP and site-specific real-time PCR. Error bars represent standard deviations determined from three independent experiments. (E) 3D FISH with probe pairs at *Hoxb* locus (*Hoxb1* and *Hoxb13*) in PFA-fixed nuclei of *Ring1A^−/−^; Ring1B^fl/fl^; R26::CreERT2* ESC lines stably expressing mock, WT, I53S, or I53A Ring1B before (−) or after (+) OHT treatment (day 2). Scale bars indicate 1 µm. The boxes show the median and interquartile range of interprobe distances (µm) in the indicated cells. Open circles indicate outliers. The statistical significance of differences between the indicated two data was examined by the Mann-Whitney U test.

Above data suggest a potential importance of H2AK119u1 for repression of key developmental regulators which is required to maintain the undifferentiated status of ESCs. This however does not necessarily prove the importance of the E3 ligase activity and H2AK119u1 *per se* for the repression because H2A ubiquitination independent functions of PRC1 in chromatin compaction and gene silencing both in vitro [24], and in vivo [23] has been reported in previous studies. To investigate this question, we expressed mutant Ring1B proteins that are defective in the interaction with the E2 component in *Ring1A/B*-dKO ESCs. In this experimental setup, we have first stably expressed exogenous WT or mutant Ring1B in *Ring1A^−/−^;Ring1B^fl/fl^;R26::CreERT2* ESCs and then endogenous Ring1B was depleted by OHT treatment (Figure 2A). Similar experiments have been described previously [23] but have made use of *Ring1B* single KO ESCs, and therefore could not exclude the contribution of low levels of Ring1A (Figure S6A) and associated H2AK119u1 that occur in ESCs [5], [17], [20], [25]. We thus tested the role of Ring1A to mediate H2AK119u1 and repression of Polycomb targets in ESCs. We first compared global H2AK119u1 levels in *Ring1B*-KO ESCs with *Ring1A/B*-dKO and found significant amount of H2AK119u1 remained in *Ring1B*-KO (Figure S6B). Consistently, the expression of exogenous Ring1A obviously restored global H2AK119u1 level, ESC identity, and repression of TP genes in *Ring1A/B*-dKO ESCs (Figure S6B–E). We then performed ChIP-chip analysis to compare Ring1A distribution with Ring1B and found that Ring1A and Ring1B significantly shared target genes (Figure S6F, G). ChIP-qPCR analysis further confirmed Ring1A binding at promoter regions of representative TP genes such as *Hoxd11* and *Zic1* in the absence of Ring1B (Figure S6H). Importantly, binding of other PRC1 components such as Mel18 was concomitantly restored by the expression of exogenous Ring1A in *Ring1A/B*-dKO ESCs (Figure S6H). Therefore, Ring1A was shown to substitute for Ring1B functions in mediating H2AK119u1 and target gene repression. These results sufficiently justify the use of *Ring1A/B*-dKO ESCs instead of *Ring1B*-KO in following experiments.

We made use of the previously characterized I53S and I53A mutations located at the E2 UbcH5c binding surface that have been shown to affect the E3 activity of Ring1B both in vitro and in vivo [17], [23], [31]. We introduced expression vectors for flag-tagged wild-type (WT) or mutant Ring1B [Ring1B (I53S) or (I53A)] into *Ring1A^−/−^;Ring1B^fl/fl^;R26::CreERT2* ESCs (Figure 2A) and established stable transfectants that expressed exogenous Ring1B at similar level to the endogenous protein (Figure 2B). Expression of WT Ring1B restored global H2AK119u1 levels in *Ring1A/B*-dKO cells whereas Ring1B (I53S) and Ring1B (I53A) did not (Figure 2B).

We went on to check whether the levels and target binding of PRC1 could be appropriately recapitulated by exogenous wild-type or mutant Ring1B in the transfectants. Levels of other PRC1 proteins were depleted in the absence of Ring1B, presumably because complex formation stabilizes individual components [9], [26]. We found that Mel18 was clearly detectable and formed complexes with Ring1B (I53S), Ring1B (I53A) and wild-type Ring1B in the absence of endogenous Ring1 proteins in each transfectant (Figure 2C). We also confirmed that levels of Cbx2 and Phc1 were restored in these transfectants (data not shown). We next assessed the association of exogenous Ring1B with target genes in the transfectants. We used ChIP and subsequent quantitative PCR (qPCR) analysis and observed binding of Ring1B I53S or I53A to target loci. Local H2AK119u1 deposition was undetectable, confirming the impaired E3 ligase activity of the Ring1B mutant proteins (Figure 2D). We also found that Mel18 binding to these targets was considerably restored by the expression of Ring1B (I53S) or Ring1B (I53A). Finally, we tested whether condensation of *Hoxb* cluster could be recapitulated in the transfectants by using 3D FISH analysis with probes for *Hoxb1* and *Hoxb13*. Consistent with a previous report using *Ring1B*-KO ESCs, we found that *Hoxb1* and *Hoxb13* were considerably separated in *Ring1A/B*-dKO ESCs compared to *Ring1A^−/−^* cells (Figure 2E) [23] and that condensation of the *Hoxb* cluster was significantly restored by the expression of Ring1B (I53S) or Ring1B (I53A). Taken together, the expression and target binding of PRC1 were sufficiently recapitulated in *Ring1A/B*-dKO ESCs that express catalytically inactive Ring1B. We thus concluded that these transfectants were well suited to address the role of Ring1B E3 activity in the maintenance and repression of ESC Polycomb targets. The above results also imply that E3 activity of Ring1B and H2AK119u1 are dispensable for PRC1 target binding.

### E3 activity of Ring1B is required to repress Polycomb targets and maintain ESCs

**Figure 3:**
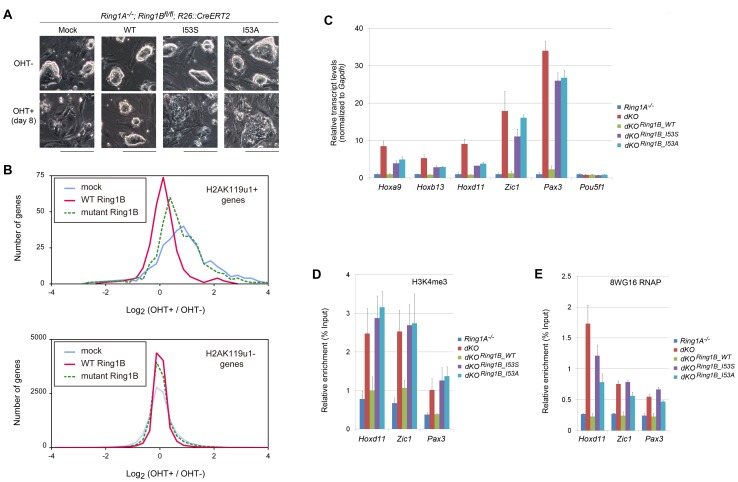
H2A ubiquitination activity of Ring1B is essential for the maintenance of ESC identity and repression of target gene expression. (A) Morphology of OHT-untreated and –treated (day 8) *Ring1A^−/−^; Ring1B^fl/fl^; R26::CreERT2* ESC lines expressing the indicated transgene. The images were acquired under a phase-contrast microscope. Scale bars indicate 200 µm. (B) Histograms showing the expression changes of H2AK119u1+ and H2AK119u1− genes in *Ring1A^−/−^; Ring1B^fl/fl^; Rosa26::CreERT2* ESCs expressing mock (blue line), WT Ring1B (red line), or mutant Ring1B (green dotted line) following OHT treatment. (C) Expression levels of *Hoxa9*, *Hoxb13*, *Hoxd11*, *Zic1*, *Pax3* and *Pou5f1* in *Ring1A^−/−^; Ring1B^fl/fl^; Rosa26::CreERT2* ESCs expressing mock, WT, I53S, or I53A Ring1B before (−) or after (+) OHT treatment (day 2). Expression levels were normalized to a *Gapdh* control and are depicted as fold changes relative to mock (OHT-untreated) ESCs. Error bars represent standard deviation determined from at least three independent experiments. (D) Local levels of trimethylated H3K4 (H3K4me3) at promoter regions of representative target genes in *Ring1A^−/−^; Ring1B^fl/fl^; R26::CreERT2* ESCs stably expressing mock, WT, I53S, or I53A Ring1B before (−) or after (+) OHT treatment (day 2) were determined by ChIP and site-specific real-time PCR. The relative amount of immunoprecipitated DNA is depicted as a percentage of input DNA. Error bars represent standard deviation determined from at least three independent experiments. (E) As in (D), but showing local levels of RNA polymerase II (RNAP) detected with the 8WG16 antibody.

We next tested the phenotypes of the transfectants after deletion of endogenous Ring1B. We observed that the expression of either Ring1B (I53S) or Ring1B (I53A) was not sufficient to maintain ESCs in an undifferentiated state (Figure 3A). We obtained similar results in the presence of three inhibitors (3i) that target FGF receptor, MEK, and GSK3 (data not shown) [32]. This implies that the E3 activity of Ring1B is required to maintain ESC identity in the absence of Ring1A. Consistently, Ring1B (I53S) failed to restore repression of differentiation markers (*Kdr*, *Gata6*, *Hnf4a* and *Cdx2*) and expression of undifferentiation markers (*Pou5f1*, *Sox2* and *Nanog*) in *Ring1A/B*-dKO ESCs while WT Ring1B or Ring1A obviously restored (Figure S7A). We went on to examine differentiation ability of the respective ESC lines by forming embryoid bodies. We found that the progressive changes in expression of the marker genes upon induction of differentiation were considerably affected in *Ring1A/B*-dKO ESCs compared to *wild-type* or *Ring1A* ^−/−^ ESCs (Figure S7B). These changes were restored by WT Ring1B but not by Ring1B (I53S). Together, Ring1B catalytic activity is required for maintenance and differentiation of ESCs.

We then examined the expression of H2AK119u1+ genes in these transfectants by using expression microarrays. In the mock transfectant, we observed that H2AK119u1+ genes were significantly de-repressed by depletion of Ring1 proteins whereas expression of H2AK119u1− genes was virtually unchanged (Figure 3B). De-repression of H2AK119u1+ genes in the *Ring1A/B*-dKO was mostly restored by the expression of WT Ring1B but only partially by Ring1B (I53S) and Ring1B (I53A) (Figure 3B; Figure S8). Moreover, the levels of restoration by Ring1B mutants were variable among target genes. To confirm the microarray results, we examined the expression of H2AK119u1 targets, *Hoxa9*, *Hoxb13*, *Hoxd11*, *Zic1*, and *Pax3*, by quantitative RT-PCR. These genes were de-repressed in *Ring1A/B*-dKO compared to OHT-untreated control cells (Figure 3C). WT Ring1B was shown to fully restore the repression of these genes. Ring1B (I53S) and Ring1B (I53A) could slightly restore the repression of *Hoxa9*, *Hoxb13* and *Hoxd11*, but almost failed to repress *Zic1* and *Pax3* (Figure 3C). Therefore, the E3 activity of Ring1B is required for efficient repression of its target genes. Our results also suggest that some genes, e.g., *Zic1* and *Pax3* are more dependent on the E3 activity than others, notably the *Hox* cluster genes such as *Hoxd11*.

### Ring1B mediates repression through H2AK119u1

The above experiments strongly suggest that repression of developmental regulators in ESCs is attributable to PRC1 mediated H2AK119u1. Previous studies report that Ring1B regulates local H3K4me3 deposition and loading of RNA polymerase II (RNAP) in ESCs [5], [19], and that H2AK119u1 has a role to suppress MLL-mediated methylation of H3 lysine 4 (H3K4) and transcriptional initiation from nucleosomal templates [28]. We, therefore, examined whether the catalytic activity of Ring1B is involved in suppressing H3K4 methylation and RNAP loading at target gene loci. Consistent with the previous reports, we found that local levels of trimethylated H3K4 (H3K4me3) and RNAP loading were considerably up-regulated at target gene promoters in *Ring1A/B*-dKO ESCs, which could be repressed by expression of WT Ring1B in these cells (Figure 3D and 3E). In contrast, Ring1B (I53S) and Ring1B (I53A) failed to suppress local increases of H3K4me3 and RNAP levels. Therefore, the catalytic activity of Ring1B is required to repress H3K4me3 and RNAP loading. Consistent with these observations, the profound reduction in local H3K27me3 levels in *Ring1A/B*-dKO ESCs could not be restored by Ring1B (I53S) or Ring1B (I53A) (Figure S9). This may also suggest the contribution of Ring1B catalytic activity to maintain repressive chromatin. Collectively, our results demonstrate that Ring1B-dependent H2AK119u1 facilitates transcriptional repression of PRC1 target genes and thereby enables the maintenance of ESC identity.

## Discussion

In the present study, we present genome-wide H2AK119u1 maps in ESCs and identify a group of genes at which H2AK119u1 is deposited in a Ring1-dependent manner. These genes are a distinctive subset of genes with H3K27me3 enrichment and we suggest that these are the central targets of Polycomb silencing to maintain ESC identity. By using mutant versions of Ring1B, which can not bind to E2 components, we demonstrate the role of H2AK119u1 to facilitate the repression of these target genes. We propose that H2AK119u1 contributes to capacitate Polycomb-mediated repression in a reversible manner because recognition and de-ubiquitination of H2AK119u1 have been shown to be linked with transcriptional activation [27], [28], [33].

This conclusion is different to a recent study which suggested that the catalytic mutant Ring1B could restore repression in *Ring1B* mutant ES cells [23]. A key difference in that study and our analyses shown here is that we assessed the function of catalytically inactive Ring1B in a background that is null for both Ring1B and the closely related homologue Ring1A. Ring1A potentially complements loss of Ring1B in ESCs, despite the fact that the expression level of Ring1A is relatively low compared to Ring1B (Figure S6) [5], [20].

Our results are concordant with those of Eskeland et al. 2010 which reports that the ability of Ring1B to mediate the condensation of the *Hoxb* cluster is not dependent on its histone ubiquitination activity. In addition, in our study we have observed that the E3 activity of Ring1B contributes to the repression of *Hox* genes to a lesser extent than to *Zic1* and *Pax3* genes (Figure 3C). Based on these evidences, we propose that H2AK119u1-dependent repression is likely complemented by other PRC1-mediated mechanisms such as chromatin compaction [23]. The fact that H2AK119u1 independent repression is more prevalent at *Hox* loci compared to other Polycomb target genes may suggest that it is more effective when target loci are closely juxtaposed *in cis*. We indeed found a slight but a significant restoration of repression of H2AK119u1+ genes that are closely juxtaposed each other (<50 kb) by expression of mutant Ring1B in *Ring1A/B*-dKO, but this is not the case for H2AK119u1+ genes that are separated by ≥50 kb genomic regions (Figure S10). However, this tendency is not statistically significant once we excluded *Hox* cluster genes. Further studies are needed to elucidate the molecular nature for H2AK119u1-independent mechanisms.

**Figure 4:**
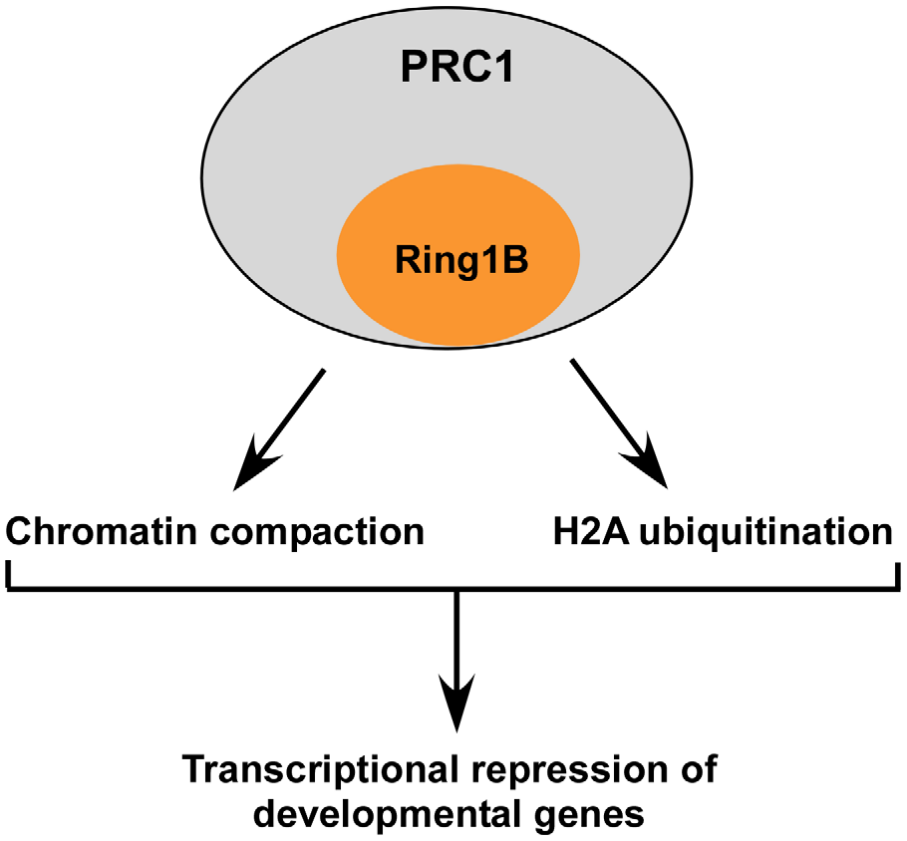
A schematic summary of this study demonstrating that PRC1-dependent repression of developmental genes in ES cells is mediated by multiple effector mechanisms.

Overall, our findings show that PRC1 mediates gene repression by combining multiple and different effector mechanisms, of which H2A ubiquitination is a major contributor (Figure 4). Such diverse PRC1 effector mechanisms might be required to make PRC1-mediated gene repression both flexible and robust. How H2A ubiquitination contributes to repress target gene transcription also awaits future studies, although mechanisms that antagonize against H2A ubiquitination have already been proposed [27].

## Materials and Methods

### Cells and cell culture

*Ring1B^fl/fl^*;*Rosa26*::*CreERT2, Ring1A^−/−^;Ring1B^fl/fl^*;*Rosa26*::*CreERT2*, and *Eed*-KO ESCs were described previously [5], [19], [20], [26], [34]. The ESCs were cultured in DMEM with 20% fetal bovine serum, MEM nonessential amino acids (Invitrogen), sodium pyruvate (Invitrogen), L-glutamine (Invitrogen), 2-mercaptoethanol (Sigma), and ESGRO (Chemicon) either on irradiated MEF as feeder layers or directly on gelatin-coated surfaces.

### Plasmids

3xFlag-tagged wild-type Ring1A, wild-type Ring1B, mutated Ring1B (I53A [17], [23] and I53S [31]) cDNAs were subcloned into the expression vector pCAG-IRES-Puro (a kind gift from Dr. Hitoshi Niwa in RIKEN CDB in Japan).

### Antibodies

The following antibodies were used: Ring1B (clone #3) [35], Ring1A [36], Phc1 [37], Mel18 (Santa Cruz; sc-10744), Cbx2 [36], H3K27me3 (Millipore; 07-449), H3K4me3 (Millipore;07-473), H2AK119u1 (E6C5; Millipore; 05-678; for ChIP), H2AK119u1 (Rabbit polyclonal; for ChIP) [28], H2AK119u1 (Rabbit polyclonal; Cell Signaling Technology; #8240; for western blot), H2A (Abcam; ab18255), RNAP (8WG16; Millipore; 05-952), Lamin B (Santa Cruz; sc-6216), mouse IgM (Millipore; 12-488), and Flag-tag (M2; Sigma; F1804).

### Stable transfection

*Ring1A^−/−^; Ring1B^fl/fl^*; *Rosa26*::*CreERT2* ESCs were stably transfected with tagged wild-type Ring1A, wild-type Ring1B, or mutated Ring1B. To establish stable transfectants, ESCs were electroporated (0.8 kV, 3 µF) with the respective expression vector and then selected for resistance to puromycin (1 µg/ml).

### Immunoprecipitation (IP) analysis

Cells expressing each of tagged constructs were suspended in IP buffer [10 mM Tris-HCl (pH8.0), 1 mM EDTA, 140 mM NaCl, 0.4% NP-40, and 0.5 mM PMSF] and sonicated for several seconds. After centrifugation, the supernatant was collected, precleared with protein G Sepharose for 30 min at 4°C, and then incubated with anti-Flag antibody (M2) for 120 min at 4°C. The immune complexes were captured by protein G Sepharose for 60 min at 4°C. The Sepharose-bound proteins were washed with IP buffer, eluted in SDS sample buffer under reducing condition, separated on SDS-PAGE gels, and subjected to western blot analysis.

### Real-time PCR

Quantitative real-time PCR was carried out with SYBR Green method and amplifications were detected with Mx3005P (Stratagene, La Jolla, CA, USA). The sequences of primers used in this study are shown in Text S1.

### Chromatin immunoprecipitation (ChIP) analysis

ESCs were treated with 1% formaldehyde/PBS for 10 min at room temperature. Cells were washed with PBS, collected and resuspended in swelling buffer [20 mM Hepes (pH 7.8), 1.5 mM MgCl_2_, 10 mM KCl, 0.1% NP-40, and 1 mM DTT] by pipetting and then kept on ice for 10 min. After Dounce homogenizing 10–20 times, the cells were centrifuged and then the pellets were resuspended in RIPA buffer [20 mM Tis-HCl (pH 8.0), 1 mM EDTA, 140 mM NaCl, 1% Triton X-100, 0.1% SDS, and 0.1% deoxycholic acid] containing protease inhibitors and sonicated into fragments with an average length of 0.3–0.5 kb. After centrifugation, the supernatants were subjected to IP with specific antibodies as previously described [19], [38]. For H2AK119u1-ChIP, pre-cleared chromatin (400 µg) was incubated with 50 µl of E6C5 antibody (overnight, 4°C) and then the chromatin-1^st^ antibody complexes were immunoprecipitated with 2^nd^ antibody (rabbit anti-mouse IgM) - preconjugated protein A dynabeads (Invitrogen). Purified immunoprecipitated and input DNA was quantified by real-time PCR, and, if necessary, was subjected to the linear amplification for ChIP-chip analysis.

### ChIP-on-chip experiment

ChIP-on-chip analysis was carried out using the Mouse Promoter ChIP-on-chip Microarray Set (G4490A, Agilent, Palo Alto, Calif., USA). ESCs were subjected to ChIP assay using specific antibodies as described in the previous section. Purified immunoprecipitated and input DNA was subjected to T7 RNA polymerase-based amplification as described previously [39]. Labeling, hybridization and washing were carried out according to the Agilent mammalian ChIP-on-chip protocol (ver.9.0). Scanned images were quantified with Agilent Feature Extraction software under standard conditions. All of experiments were performed by using at least two biological replicates. The obtained data were analyzed as described in Text S1.

### Gene expression microarray

Total RNA was extracted using the Trizol reagent (Invitrogen, Carlsbad, CA, USA) and purified with Qiagen RNeasy separation columns (Qiagen, Hilden, Germany). First strand cDNA was synthesized and hybridized to Affymetrix GeneChip Mouse Genome 430 2.0 arrays (Affymetrix, Santa Clara, CA, USA) to assess and compare the overall gene expression profiles. The obtained data were analyzed as described in Text S1.

### Three dimensional (3D)-DNA-FISH

3D-DNA-FISH with spatial preservation of chromatin architecture was performed as described previously [40]. Experimental details are described in Text S1.

### Accession numbers

ChIP-chip and microarray data discussed in this publication have been deposited in NCBI's Gene Expression Omnibus and is accessible through GEO Series accession number GSE38650.

## Supporting Information

Figure S1 Examples of the distribution of H3K27me3, Ring1B, H2AK119u1 (E6C5), H2AK119u1 (Rabbit) and H2A at the promoter regions of representative genes. Plots display log_2_ values of unprocessed ChIP-enrichment ratios for all probes within a genomic region (ChIP-enriched versus input DNA). Green plots show the results from control ESCs (OHT−; OHT-untreated). Red plots for Ring1B, H2AK119u1 and H2A show the results from *Ring1A/B*-dKO ESCs (OHT+; 2 days after OHT treatment). Yellow plots for H3K27me3 show the results from *Eed*-KO ESCs. The transcription start sites (TSSs) are denoted by arrows.
(PDF)

Figure S2 Local levels of H2AK119u1 and Ring1B at promoter regions of representative genes in *Ring1A^−/−^; Ring1B^fl/fl^; R26::CreERT2* ESCs before (−) or after (+) OHT treatment (day 2) were determined by ChIP and quantitative PCR. Those of H3K27me3 in *wild-type* and *Eed-*KO ESCs were also analyzed. Relative amount of immunoprecipitated DNA is depicted as a percentage of input DNA. Error bars represent standard deviation determined from at least three independent experiments. (B) Expression levels of representative genes in *wild-type*, *Ring1A^−/−^*, and *Ring1A/B-*dKO (2, 4, and 6 days after the start of OHT treatment) were determined by the quantitative RT-PCR. Expression levels were normalized to a *Gapdh* control and are depicted as fold changes relative to *wild-type* ESCs. Error bars represent standard deviation determined from at least three independent experiments.
(PDF)

Figure S3 (A, B) ChIP-on-chip analysis showing the average of H2AK119u1 (E6C5) (A) and H2A (B) distributions at the promoter regions (from −5 kb to +5 kb relative to TSS) of Ring1B-bound and –unbound genes in *Ring1A^−/−^* (OHT−; green line) and *Ring1A/B*-dKO (OHT+; red line) ESCs. Enrichment of H2AK119u1 and H2A is expressed relative to input DNA. (C) Detection of ChIP-chip positive genes using approximated Gaussian distributions. Geometric mean of Ring1B and H2AK119u1 enrichment for each gene in *Ring1A/B*-dKO ESCs (OHT+) was subtracted from that in *Ring1A^−/−^* ESCs (OHT−) to provide histograms showing the distribution of *Ring1B* depletion-sensitive enrichment of Ring1B (green) and H2AK119u1 (E6C5; orange, rabbit polyclonal; brown). Geometric mean of H3K27me3 enrichment in *Eed*-KO ESCs for each gene was subtracted from that in *wild-type* ESCs to provide a histogram showing the distribution of PRC2 deficiency-sensitive enrichment of H3K27me3 (blue). Each histogram was approximated using two Gaussian distributions (pink), and the mean +3sd value of the lower distribution was used as a threshold to determine positive genes (dotted blue). (D) Venn diagram representing the overlap of H2AK119u1 target genes identified by using two different antibodies (E6C5 and rabbit polyclonal, respectively). Numbers in parentheses represent the total number of genes occupied by each one. The probability of the overlap between these target genes is calculated and shown (*P*). (E) Venn diagram representing the overlap of H3K27me3 target genes identified in this and a previous ChIP-seq study (Mikkelsen TS et al., Nature. 2007). The probability of the overlap between these target genes is calculated and shown (*P*). (F) As in (E), but showing the overlap of Ring1B-bound genes identified in this and a previous ChIP-seq study [Ku M, Koche RP, Rheinbay E, Mendenhall EM, Endoh M, et al. (2008) Genomewide Analysis of PRC1 and PRC2 Occupancy Identifies Two Classes of Bivalent Domains. PLoS Genet 4(10): e1000242. doi:10.1371/journal.pgen.1000242].
(PDF)

Figure S4 Scatter plots demonstrating the overall correlation of occupancy levels between H3K27me3 and Ring1B for each gene. The geometric mean of H3K27me3 and Ring1B enrichment for each gene is depicted and Pearson's correlation efficient (*r*) was calculated. H2AK119u1-positive and –negative genes are depicted as orange and blue dots, respectively.
(PDF)

Figure S5 Gene ontology (GO) term analysis showing that genes related to transcription and/or development are highly over-represented among H2AK119u1-positive genes. H3K27me3-positive genes were classified according to the presence (+) or absence (−) of Ring1B and H2AK119u1 and the enrichment of respective GO terms in each subset of genes was calculated. The percentages of respective subsets of genes in a particular GO group are graphed along the right half of the x-axis, and *p*-values for the significance of over- or under-representation against total genes are graphed along the left half of the x-axis. Significant under-representation is indicated by asterisks beside the respective *p*-value bars.
(PDF)

Figure S6 (A) Expression levels of *Ring1A* in *wild-type*, *Ring1B^−/−^*, and *Ring1A^−/−^* ESCs were determined by the quantitative RT-PCR. Expression levels were normalized to a *Gapdh* control and are depicted as fold over *wild-type* ESCs. Error bars represent standard deviation determined from at least three independent experiments. (B) Immunoblot analysis of Ring1B, Ring1A, Flag, H2AK119u1 and Lamin B protein levels in whole cell lysates of *wild-type*, *Ring1B^−/−^*, and *Ring1A^−/−^; Ring1B^fl/fl^; R26::CreERT2* ESC lines expressing mock or Flag-tagged Ring1A construct with or without OHT treatment (OHT+ and −, respectively). The locations of bands of endogenous and exogenous Ring1A are indicated by arrow heads. No sample was loaded on the lane indicated by an arrow. (C) Graph showing proliferation of the indicated ESC lines after OHT treatment. OHT-treated *Ring1A^−/−^; Ring1B^fl/fl^; R26::CreERT2* ESC lines stably expressing mock or Flag-tagged Ring1A construct (2 transfectants; #1 & #4) were indicated as *dKO* and *dKO^Ring1A^*, respectively. (D) Morphology of the indicated ESC lines. The images were acquired under a phase-contrast microscope. Scale bars indicate 200 µm. (E) Expression levels of *Hoxa9*, *Hoxd11*, *Zic1* and *Pax3* in *wild-type*, *Ring1A^−/−^* and *Ring1A/B*-dKO ESCs expressing mock or Flag-tagged Ring1A construct (2 days after the start of OHT treatment) were determined by the quantitative RT-PCR. Expression levels were normalized to a *Gapdh* control and are depicted as fold over *Ring1A^−/−^* ESCs. Error bars represent standard deviation determined from at least three independent experiments. (F) Venn diagram representing the overlap among genes occupied by Ring1B and Ring1A. Ring1B- and Flag-Ring1A-bound genes were determined by ChIP-on-chip experiments using Ring1B and Flag antibodies. Numbers in parentheses represent the total number of genes occupied by each one. (G) ChIP-on-chip analysis showing the average of 3xFlag-Ring1A distributions at the promoter regions (from −6 kb to +6 kb relative to TSS) in *Ring1A^−/−^; Ring1B^fl/fl^; R26::CreERT2* ESCs with (OHT+ day2: green dotted line) or without (OHT−: orange line) OHT treatment. Enrichment of Flag-tagged Ring1A is expressed relative to input DNA. (H) Local levels of H2AK119u1, Mel18, Flag-tagged Ring1A and Ring1B at promoter regions of *Hoxd11* and *Zic1* in the indicated ESC lines were determined by ChIP and quantitative PCR. Error bars represent standard deviations determined from three independent experiments.
(PDF)

Figure S7 (A) Expression levels of undifferentiation and differentiation markers in *wild-type*, *Ring1A^−/−^*, and *Ring1A/B-dKO* ESCs (2, 4, or 6 days after the start of OHT treatment) expressing mock, WT Ring1B, I53S Ring1B, or Ring1A construct were investigated by the quantitative RT-PCR. Expression levels were normalized to a *Gapdh* control and are depicted as fold changes relative to the *wild-type* ESCs. Error bars represent standard deviation determined from at least three independent experiments. (B) Expression levels of the indicated markers in w*ild-type*, *Ring1A^−/−^*, and *Ring1A/B-dKO* ESCs expressing mock, WT Ring1B, or I53S Ring1B construct cultured in differentiation condition for the indicated days were investigated by the quantitative RT-PCR. We treated *Ring1A^−/−^; Ring1B^fl/fl^; R26::CreERT2* ESC lines stably expressing mock, WT Ring1B, or I53S Ring1B construct with OHT for 2 days to generate *Ring1A/B-dKO* ESCs expressing either of the constructs. Then, *wild-type*, *Ring1A^−/−^*, and these OHT-treated ESCs were subjected to embryoid body formation and cultured for 3, 6, or 9 days in the absence of LIF and feeder cells. Expression levels were normalized to a *Gapdh* control and are depicted as fold changes relative to the undifferentiated *wild-type* ESCs. Error bars represent standard deviation determined from at least three independent experiments.
(PDF)

Figure S8 A heat map with hierarchical clustering showing de-repressed (green), unchanged (black), or repressed (red) H2AK119u1+ genes upon OHT treatment (day 2) in *Ring1A^−/−^*; *Ring1B^fl/fl^*; *Rosa26::CreERT2* ESCs stably expressing mock, WT, I53S, or I53A Ring1B construct was generated from the microarray data.
(PDF)

Figure S9 Local levels of H3K27me3 at promoter regions of the representative target genes in *wild-type* and *Ring1A^−/−^; Ring1B^fl/fl^; R26::CreERT2* ESCs stably expressing mock, WT, I53S, or I53A Ring1B construct before (−) or after (+) OHT treatment (day 2) were determined by ChIP and site-specific real-time PCR. The relative amount of immunoprecipitated DNA is depicted as a percentage of input DNA. Error bars represent standard deviation determined from at least three independent experiments.
(PDF)

Figure S10 We arbitrarily divided H2AK119u1-positive genes into three groups based on the intergenic distances to the closest H2AK119u1-positive genes, and compared the level of de-repression upon OHT treatment in *Ring1A^−/−^*; *Ring1B^fl/fl^*; *Rosa26::CreERT2* ESCs expressing mock, wild-type, or mutant Ring1B construct using the microarray data. The distance between each H2AK119u1-positive gene was determined using the annotation of the reference mouse genome (NCBI version 36, mm8). The boxes show the median and interquartile range of the expression changes upon OHT treatment. Open circles indicate outliers. The differences of the expression changes between the indicated two groups were statistically evaluated using Mann-Whitney's U-test, because the numbers of applied genes were too small to expect normal distribution.
(PDF)

Table S1 Mean ChIP-chip enrichment score for each gene. Geometric mean of Ring1B and H2AK119u1 enrichment for each gene in *Ring1A/B*-dKO ESCs (OHT+) was subtracted from that in *Ring1A^−/−^* ESCs (OHT−) to provide *Ring1B* depletion-sensitive enrichment of Ring1B and H2AK119u1. Geometric mean of H3K27me3 enrichment in *Eed*-KO ESCs for each gene was subtracted from that in *wild-type* ESCs to provide PRC2 deficiency-sensitive enrichment of H3K27me3. The threshold between signal and noise in each ChIP-chip experiment was determined as described in Figure S3C, and the enrichment scores that are more than the threshold value are depicted as red.
(XLS)

Text S1 Supporting Methods.
(DOC)
