# Supplementary figures and images for: Histone H2A Mono-Ubiquitination Is a Crucial Step to Mediate PRC1-Dependent Repression of Developmental Genes to Maintain ES Cell Identity
Source: PLoS Genet. 2012 Jul 26;8(7):e1002774. doi: 10.1371/journal.pgen.1002774 (PMC3405999; doi:10.1371/journal.pgen.1002774)

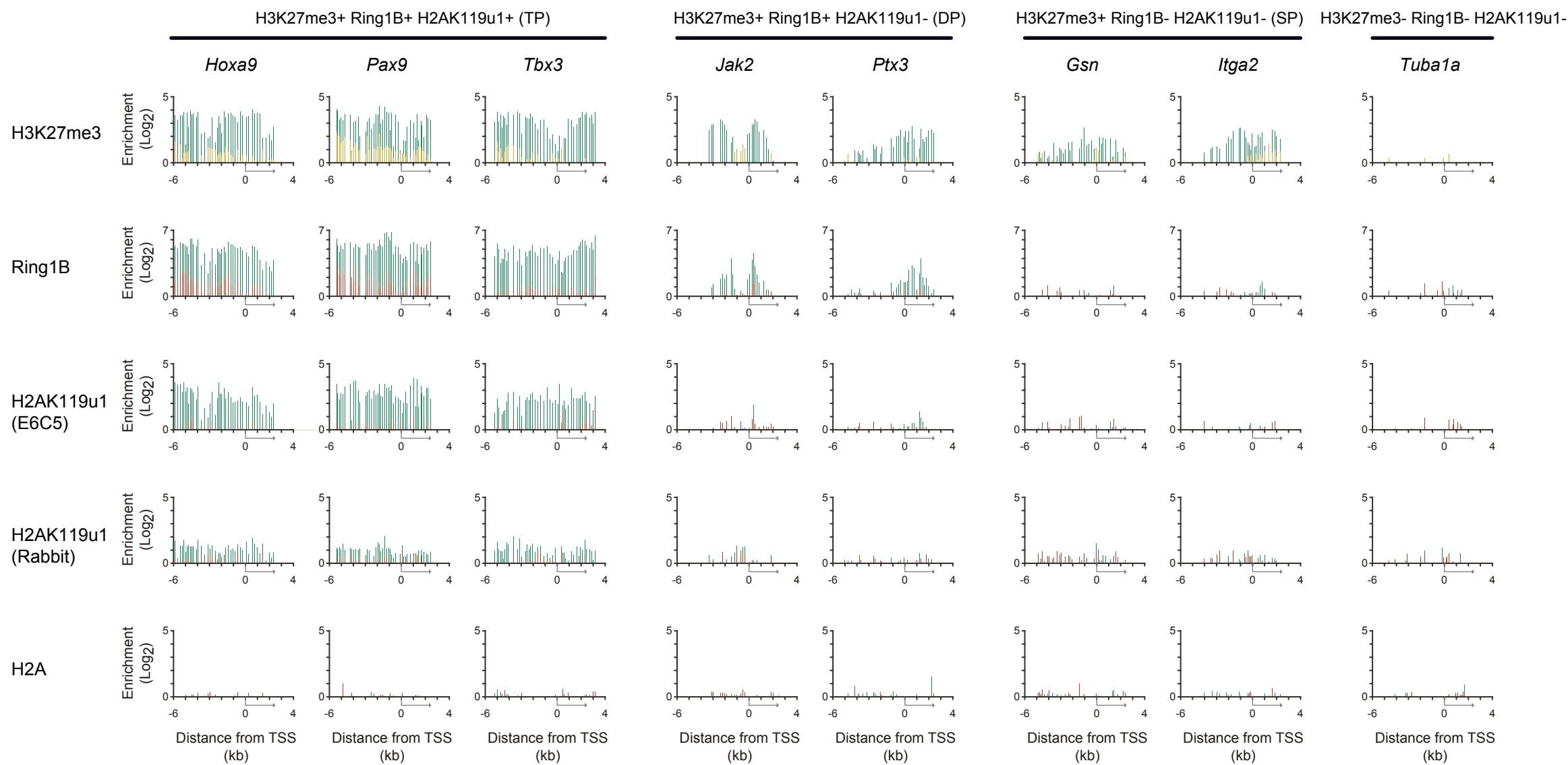

Supplement: Figure S1 — Examples of the distribution of H3K27me3, Ring1B, H2AK119u1 (E6C5), H2AK119u1 (Rabbit) and H2A at the promoter regions of representative genes. Plots display log2 values of unprocessed ChIP-enrichment ratios for all probes within a genomic region (ChIP-enriched versus input DNA). Green plots show the results from control ESCs (OHT−; OHT-untreated). Red plots for Ring1B, H2AK119u1 and H2A show the results from Ring1A/B-dKO ESCs (OHT+; 2 days after OHT treatment). Yellow plots for H3K27me3 show the results from Eed-KO ESCs. The transcription start sites (TSSs) are denoted by arrows. (PDF) [file pgen.1002774.s001.pdf]

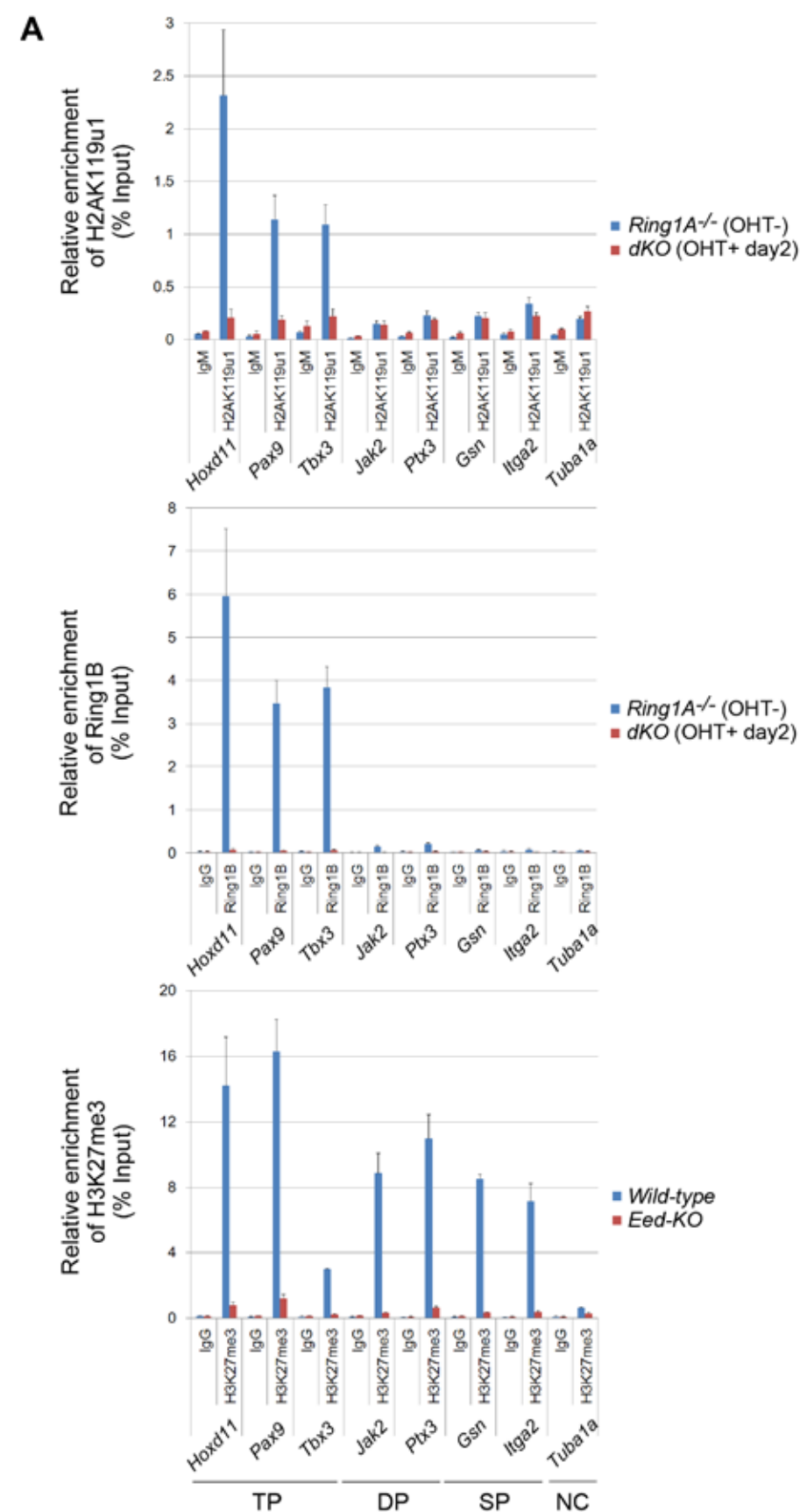

**B**

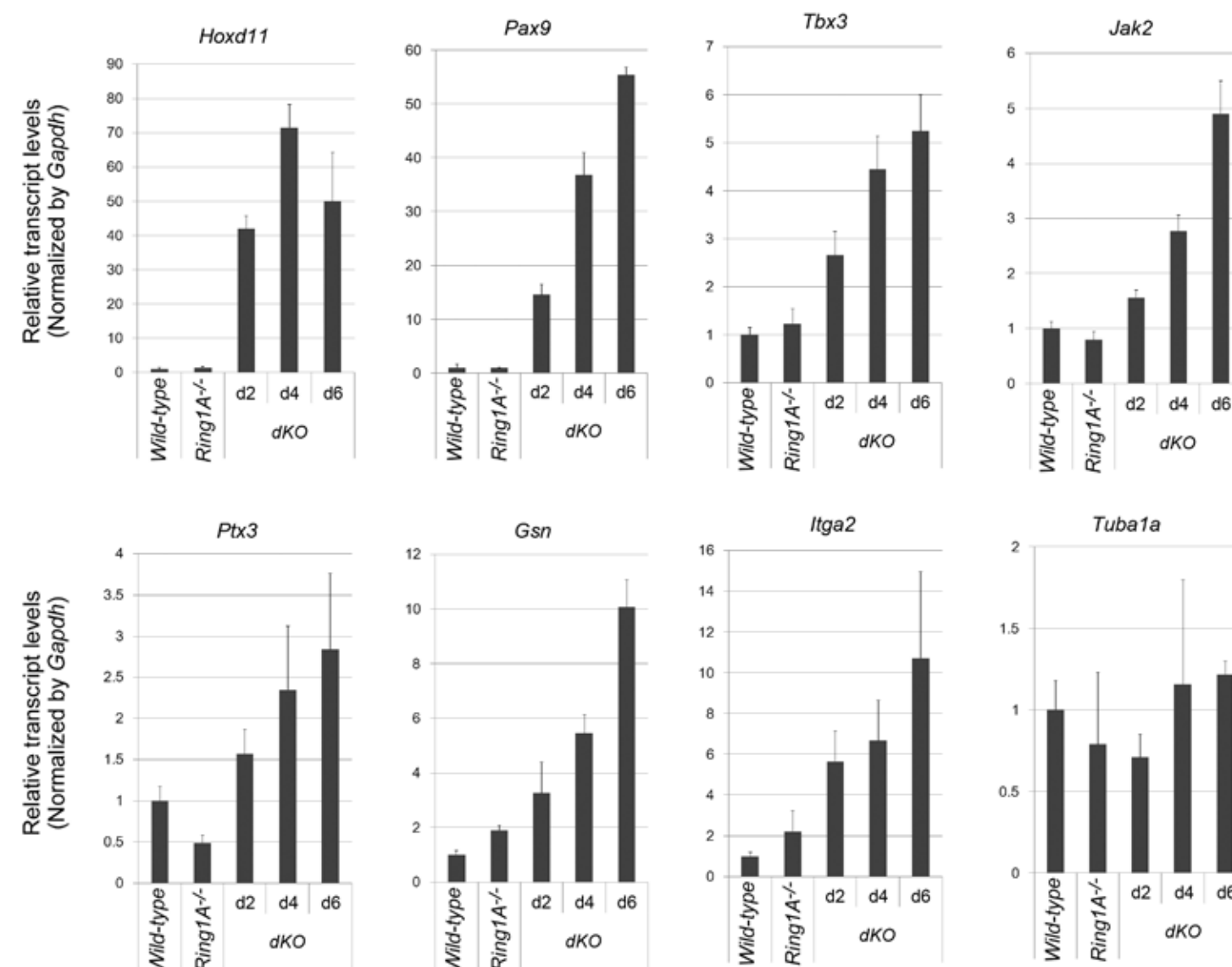

Supplement: Figure S2 — Local levels of H2AK119u1 and Ring1B at promoter regions of representative genes in Ring1A−/−; Ring1Bfl/fl; R26::CreERT2 ESCs before (−) or after (+) OHT treatment (day 2) were determined by ChIP and quantitative PCR. Those of H3K27me3 in wild-type and Eed-KO ESCs were also analyzed. Relative amount of immunoprecipitated DNA is depicted as a percentage of input DNA. Error bars represent standard deviation determined from at least three independent experiments. (B) Expression levels of representative genes in wild-type, Ring1A−/−, and Ring1A/B-dKO (2, 4, and 6 days after the start of OHT treatment) were determined by the quantitative RT-PCR. Expression levels were normalized to a Gapdh control and are depicted as fold changes relative to wild-type ESCs. Error bars represent standard deviation determined from at least three independent experiments. (PDF) [file pgen.1002774.s002.pdf]

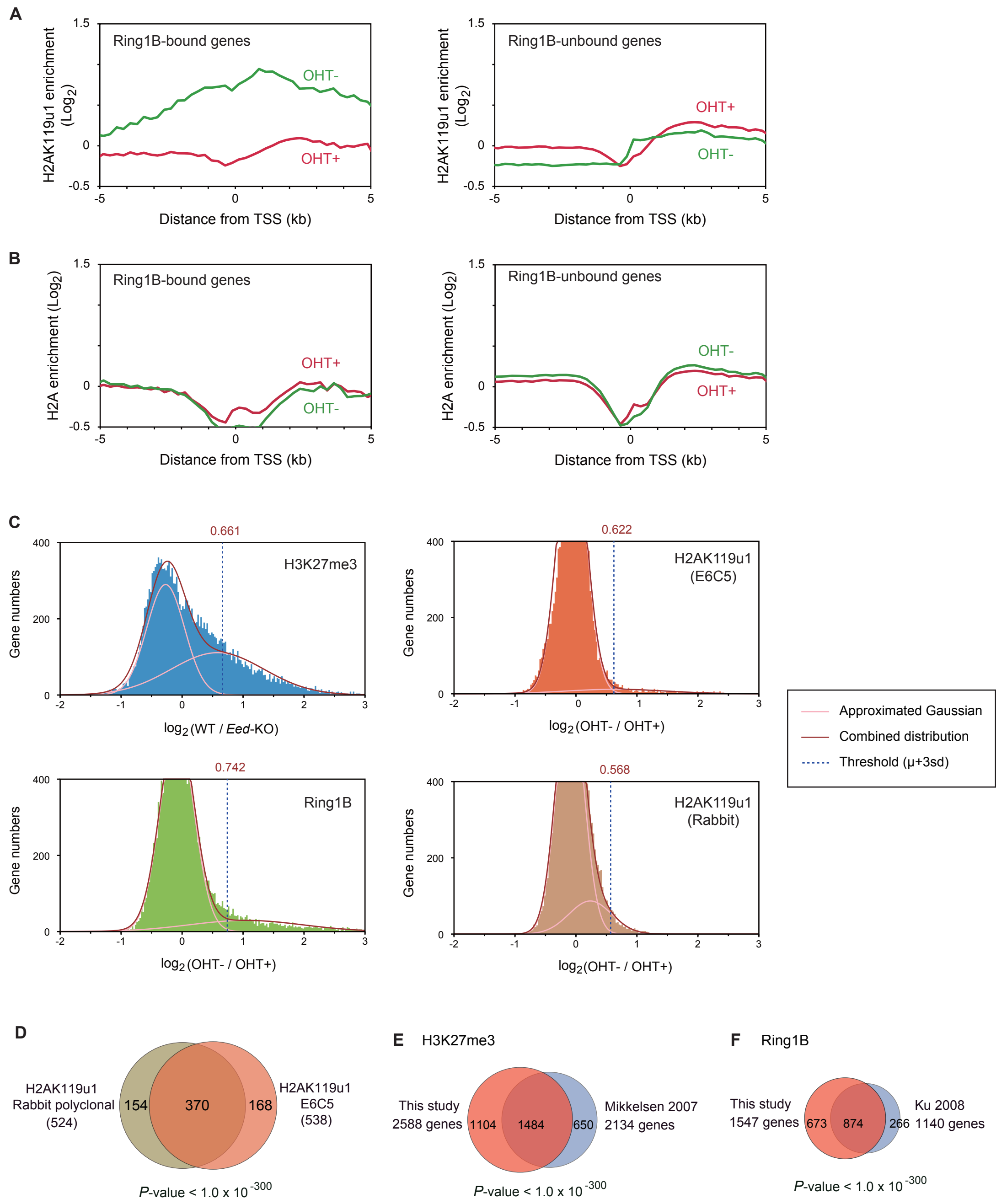

Supplement: Figure S3 — (A, B) ChIP-on-chip analysis showing the average of H2AK119u1 (E6C5) (A) and H2A (B) distributions at the promoter regions (from −5 kb to +5 kb relative to TSS) of Ring1B-bound and –unbound genes in Ring1A−/− (OHT−; green line) and Ring1A/B-dKO (OHT+; red line) ESCs. Enrichment of H2AK119u1 and H2A is expressed relative to input DNA. (C) Detection of ChIP-chip positive genes using approximated Gaussian distributions. Geometric mean of Ring1B and H2AK119u1 enrichment for each gene in Ring1A/B-dKO ESCs (OHT+) was subtracted from that in Ring1A−/− ESCs (OHT−) to provide histograms showing the distribution of Ring1B depletion-sensitive enrichment of Ring1B (green) and H2AK119u1 (E6C5; orange, rabbit polyclonal; brown). Geometric mean of H3K27me3 enrichment in Eed-KO ESCs for each gene was subtracted from that in wild-type ESCs to provide a histogram showing the distribution of PRC2 deficiency-sensitive enrichment of H3K27me3 (blue). Each histogram was approximated using two Gaussian distributions (pink), and the mean +3sd value of the lower distribution was used as a threshold to determine positive genes (dotted blue). (D) Venn diagram representing the overlap of H2AK119u1 target genes identified by using two different antibodies (E6C5 and rabbit polyclonal, respectively). Numbers in parentheses represent the total number of genes occupied by each one. The probability of the overlap between these target genes is calculated and shown (P). (E) Venn diagram representing the overlap of H3K27me3 target genes identified in this and a previous ChIP-seq study (Mikkelsen TS et al., Nature. 2007). The probability of the overlap between these target genes is calculated and shown (P). (F) As in (E), but showing the overlap of Ring1B-bound genes identified in this and a previous ChIP-seq study [Ku M, Koche RP, Rheinbay E, Mendenhall EM, Endoh M, et al. (2008) Genomewide Analysis of PRC1 and PRC2 Occupancy Identifies Two Classes of Bivalent Domains. PLoS Genet 4(10): e1000242. doi:10 [file pgen.1002774.s003.pdf]

Endoh et al.  
Figure S4

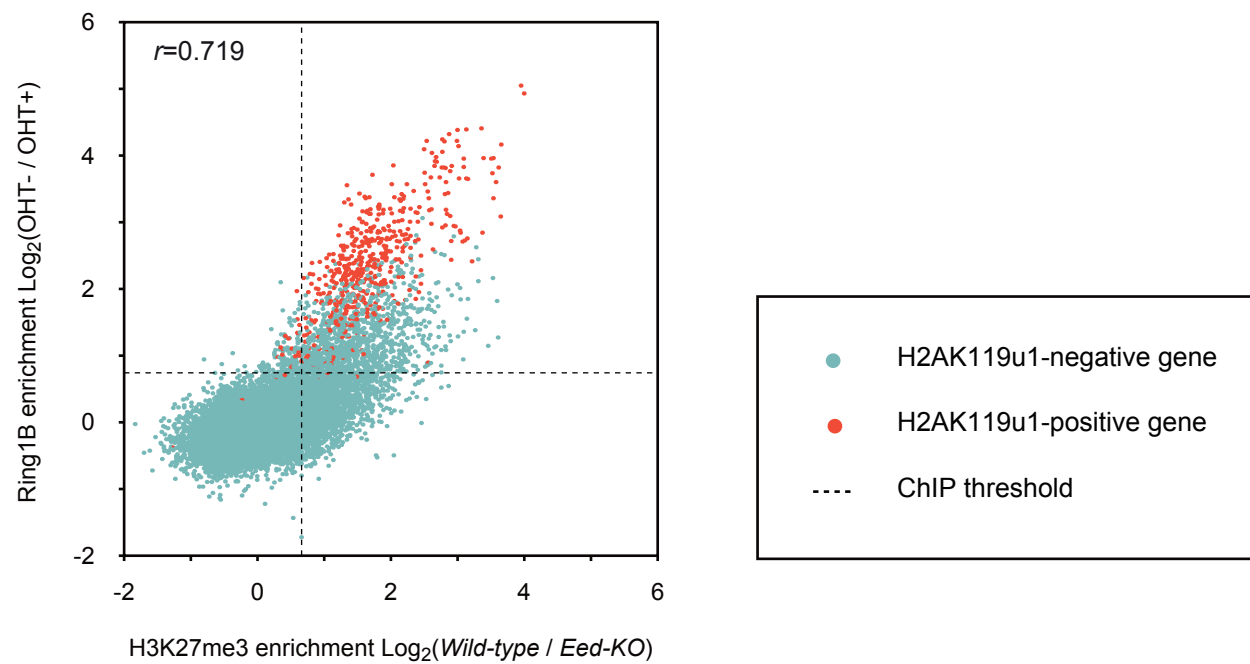

Supplement: Figure S4 — Scatter plots demonstrating the overall correlation of occupancy levels between H3K27me3 and Ring1B for each gene. The geometric mean of H3K27me3 and Ring1B enrichment for each gene is depicted and Pearson's correlation efficient (r) was calculated. H2AK119u1-positive and –negative genes are depicted as orange and blue dots, respectively. (PDF) [file pgen.1002774.s004.pdf]

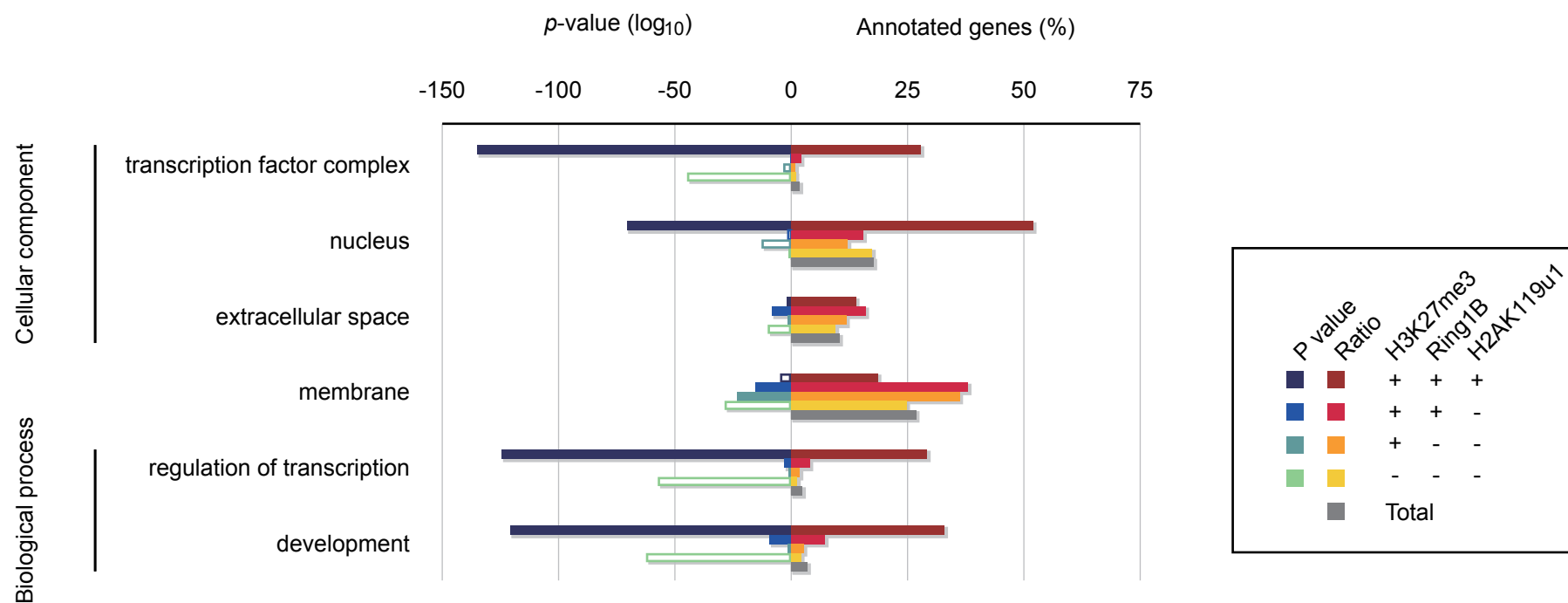

Supplement: Figure S5 — Gene ontology (GO) term analysis showing that genes related to transcription and/or development are highly over-represented among H2AK119u1-positive genes. H3K27me3-positive genes were classified according to the presence (+) or absence (−) of Ring1B and H2AK119u1 and the enrichment of respective GO terms in each subset of genes was calculated. The percentages of respective subsets of genes in a particular GO group are graphed along the right half of the x-axis, and p-values for the significance of over- or under-representation against total genes are graphed along the left half of the x-axis. Significant under-representation is indicated by asterisks beside the respective p-value bars. (PDF) [file pgen.1002774.s005.pdf]

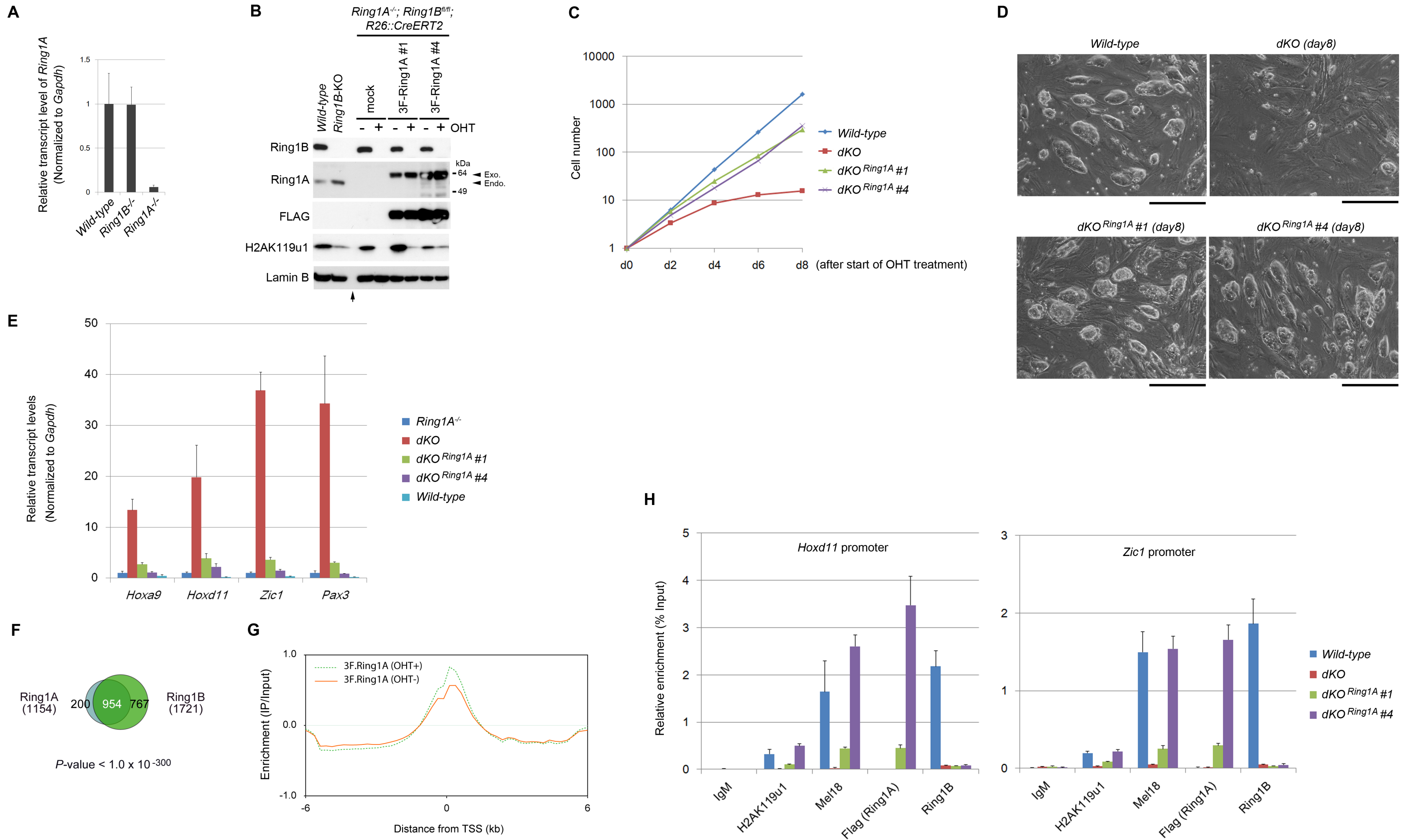

Supplement: Figure S6 — (A) Expression levels of Ring1A in wild-type, Ring1B−/−, and Ring1A−/− ESCs were determined by the quantitative RT-PCR. Expression levels were normalized to a Gapdh control and are depicted as fold over wild-type ESCs. Error bars represent standard deviation determined from at least three independent experiments. (B) Immunoblot analysis of Ring1B, Ring1A, Flag, H2AK119u1 and Lamin B protein levels in whole cell lysates of wild-type, Ring1B−/−, and Ring1A−/−; Ring1Bfl/fl; R26::CreERT2 ESC lines expressing mock or Flag-tagged Ring1A construct with or without OHT treatment (OHT+ and −, respectively). The locations of bands of endogenous and exogenous Ring1A are indicated by arrow heads. No sample was loaded on the lane indicated by an arrow. (C) Graph showing proliferation of the indicated ESC lines after OHT treatment. OHT-treated Ring1A−/−; Ring1Bfl/fl; R26::CreERT2 ESC lines stably expressing mock or Flag-tagged Ring1A construct (2 transfectants; #1 & #4) were indicated as dKO and dKORing1A, respectively. (D) Morphology of the indicated ESC lines. The images were acquired under a phase-contrast microscope. Scale bars indicate 200 µm. (E) Expression levels of Hoxa9, Hoxd11, Zic1 and Pax3 in wild-type, Ring1A−/− and Ring1A/B-dKO ESCs expressing mock or Flag-tagged Ring1A construct (2 days after the start of OHT treatment) were determined by the quantitative RT-PCR. Expression levels were normalized to a Gapdh control and are depicted as fold over Ring1A−/− ESCs. Error bars represent standard deviation determined from at least three independent experiments. (F) Venn diagram representing the overlap among genes occupied by Ring1B and Ring1A. Ring1B- and Flag-Ring1A-bound genes were determined by ChIP-on-chip experiments using Ring1B and Flag antibodies. Numbers in parentheses represent the total number of genes occupied by each one. (G) ChIP-on-chip analysis showing the average of 3xFlag-Ring1A distributions at the promoter regions (from −6 kb to +6 kb relative to TSS) [file pgen.1002774.s006.pdf]

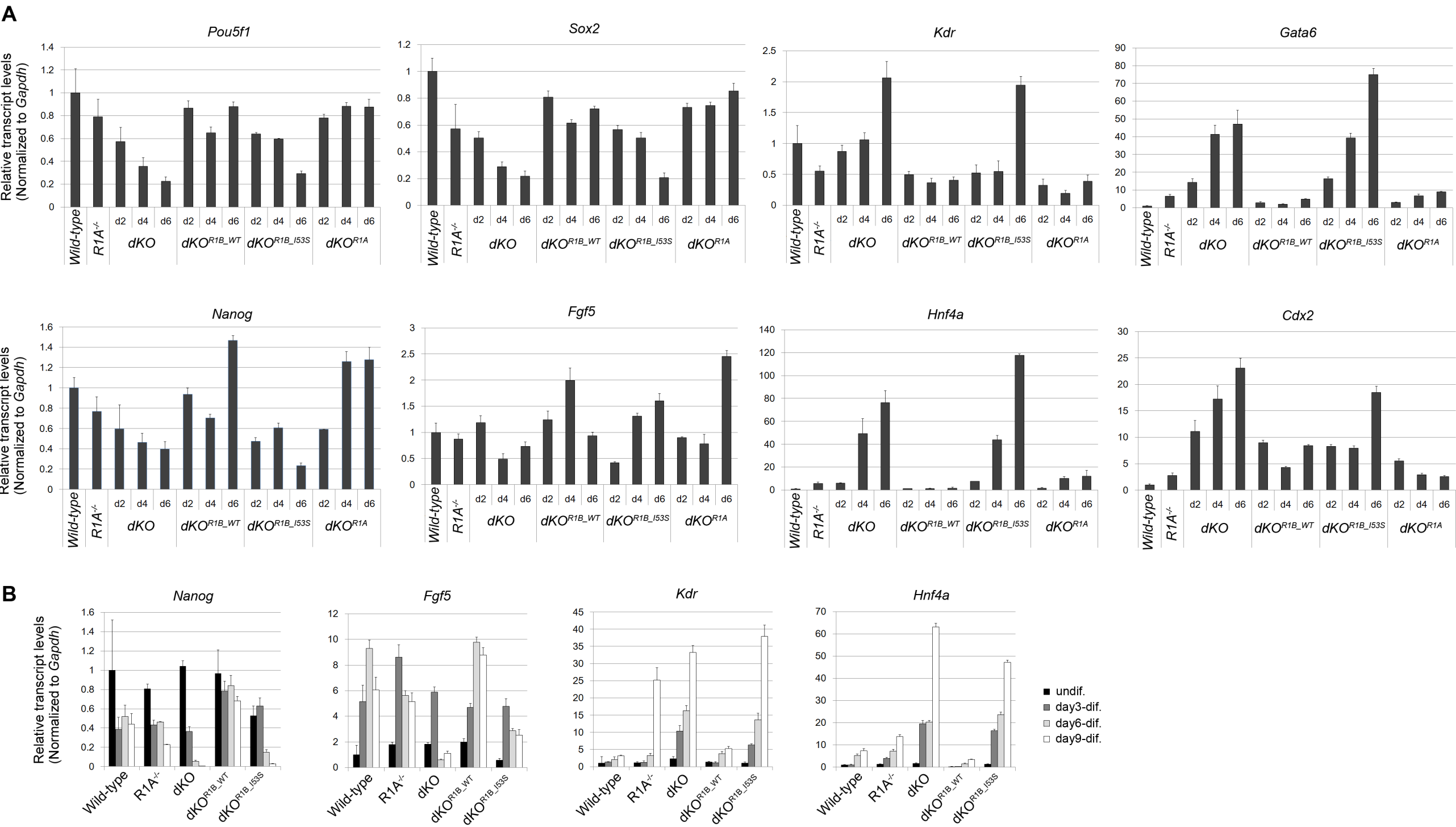

Supplement: Figure S7 — (A) Expression levels of undifferentiation and differentiation markers in wild-type, Ring1A−/−, and Ring1A/B-dKO ESCs (2, 4, or 6 days after the start of OHT treatment) expressing mock, WT Ring1B, I53S Ring1B, or Ring1A construct were investigated by the quantitative RT-PCR. Expression levels were normalized to a Gapdh control and are depicted as fold changes relative to the wild-type ESCs. Error bars represent standard deviation determined from at least three independent experiments. (B) Expression levels of the indicated markers in wild-type, Ring1A−/−, and Ring1A/B-dKO ESCs expressing mock, WT Ring1B, or I53S Ring1B construct cultured in differentiation condition for the indicated days were investigated by the quantitative RT-PCR. We treated Ring1A−/−; Ring1Bfl/fl; R26::CreERT2 ESC lines stably expressing mock, WT Ring1B, or I53S Ring1B construct with OHT for 2 days to generate Ring1A/B-dKO ESCs expressing either of the constructs. Then, wild-type, Ring1A−/−, and these OHT-treated ESCs were subjected to embryoid body formation and cultured for 3, 6, or 9 days in the absence of LIF and feeder cells. Expression levels were normalized to a Gapdh control and are depicted as fold changes relative to the undifferentiated wild-type ESCs. Error bars represent standard deviation determined from at least three independent experiments. (PDF) [file pgen.1002774.s007.pdf]

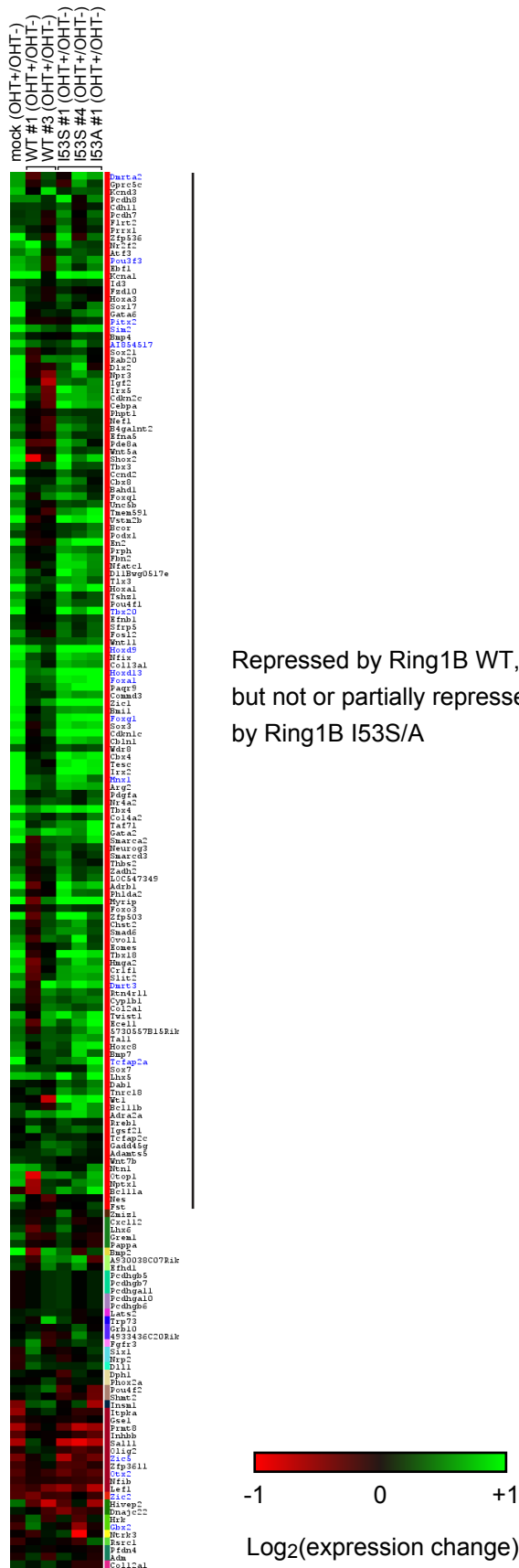

Supplement: Figure S8 — A heat map with hierarchical clustering showing de-repressed (green), unchanged (black), or repressed (red) H2AK119u1+ genes upon OHT treatment (day 2) in Ring1A−/−; Ring1Bfl/fl; Rosa26::CreERT2 ESCs stably expressing mock, WT, I53S, or I53A Ring1B construct was generated from the microarray data. (PDF) [file pgen.1002774.s008.pdf]

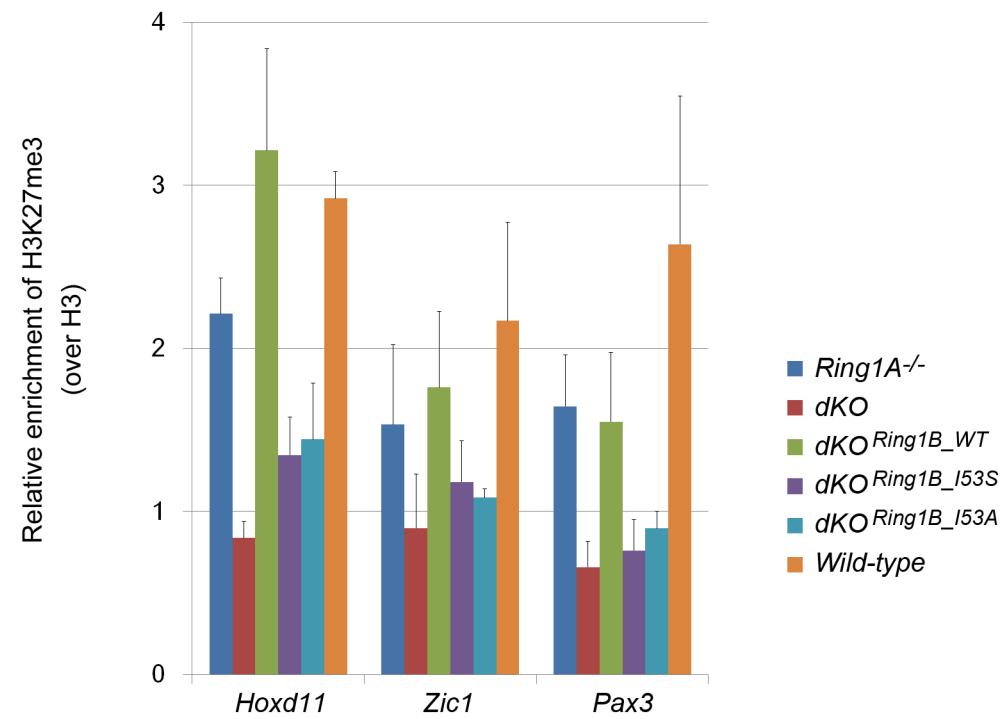

Supplement: Figure S9 — Local levels of H3K27me3 at promoter regions of the representative target genes in wild-type and Ring1A−/−; Ring1Bfl/fl; R26::CreERT2 ESCs stably expressing mock, WT, I53S, or I53A Ring1B construct before (−) or after (+) OHT treatment (day 2) were determined by ChIP and site-specific real-time PCR. The relative amount of immunoprecipitated DNA is depicted as a percentage of input DNA. Error bars represent standard deviation determined from at least three independent experiments. (PDF) [file pgen.1002774.s009.pdf]

Endoh et al.  
Figure S10

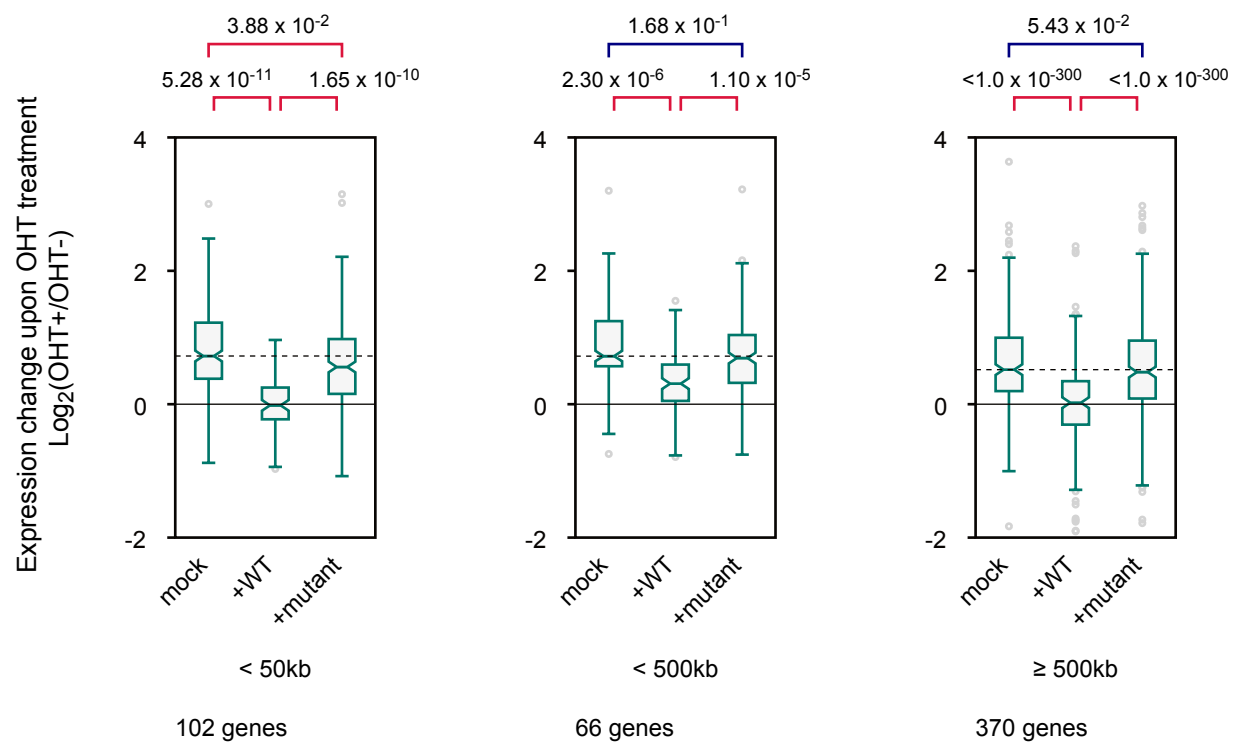

Supplement: Figure S10 — We arbitrarily divided H2AK119u1-positive genes into three groups based on the intergenic distances to the closest H2AK119u1-positive genes, and compared the level of de-repression upon OHT treatment in Ring1A−/−; Ring1Bfl/fl; Rosa26::CreERT2 ESCs expressing mock, wild-type, or mutant Ring1B construct using the microarray data. The distance between each H2AK119u1-positive gene was determined using the annotation of the reference mouse genome (NCBI version 36, mm8). The boxes show the median and interquartile range of the expression changes upon OHT treatment. Open circles indicate outliers. The differences of the expression changes between the indicated two groups were statistically evaluated using Mann-Whitney's U-test, because the numbers of applied genes were too small to expect normal distribution. (PDF) [file pgen.1002774.s010.pdf]
