# Supplementary material for: Histone H2A Mono-Ubiquitination Is a Crucial Step to Mediate PRC1-Dependent Repression of Developmental Genes to Maintain ES Cell Identity
Source: PLoS Genet. 2012 Jul 26;8(7):e1002774. doi: 10.1371/journal.pgen.1002774 (PMC3405999; doi:10.1371/journal.pgen.1002774)
Supplement: Text S1 — Supporting Methods. (DOC) [file pgen.1002774.s012.doc]

**Method S1**

**The sequences of primers used in quantitative ChIP-PCR and RT-PCR**

[For ChIP-qPCR]

*Hoxd11* Forward primer: CACTCTTGTCCCTGGTGTCA

Reverse primer: CTGGGAGCTTGTTGCTTCTT

*Pax3* Forward primer: GGGTGTAAGGGAGGAGTGTTC

Reverse primer: GGATATGACGCCAGGAGTGT

*Pax9* Forward primer: GCTTCCTCGCCATCATACAT

Reverse primer: ATACTCCTGGCTGAGGGACA

*Zic1* Forward primer: CAAACCTCAGGAACCAGGAA

Reverse primer: TGGTGTCTTTCACAGCCTCA

*Tbx3* Forward primer: CGCAGGAGCTAGAGGATCTG

Reverse primer : CGGCCAATAAGCCTTAACAA

*Jak2* Forward primer: CCGTTACTGGCAATCACACA

Reverse primer: CAAAGAGCCACGTTCATTCA

*Ptx3* Forward primer: CGAGTGCTCACACCTTTGAA

Reverse primer: AAGCAGGATTTGGAACGAGA

*Gsn* Forward primer: CCAGGGAATATTTGCCTTCA

Reverse primer: TCTGACATGTCCAGCCTACG

*Itga2* Forward primer: AGCAGAGAAGGGCACACACT

Reverse primer: GATCCACCCACAACCAACAT

*Tuba1a* Forward primer: CTCTGACTCCCAGGTGCTGT

Reverse primer: GGCGTGGCTGAATGTTTAGT

[For RT-qPCR]

*Hoxa9* Forward primer: GCTTGTGGTTCTCCTCCAGT

Reverse primer: AGTTCCAGCGTCTGGTGTTT

*Hoxb13* Forward primer: CCTATAGCAAGGGGCAGTTG

Reverse primer: GAGATCTTGCGCCTCTTGTC

*Hoxd11* Forward primer: CTCCAACTCTCTCGGATGCT

Reverse primer: CAGACGGTCCCTGTTCAGTT

*Pax3* Forward primer: GCCGACCCTGCCAA CATAC

Reverse primer: TGCTTTGGTGTACAGTGCTCG

*Pax9* Forward primer: TTGGAGCAGGAAGCCAAGTA

Reverse primer: ACCAGAAGGAGCAGCACTGT­

*Zic1* Forward primer: CTTTTCCCTGCCCGTTTC

Reverse primer: CTCGAACTCGCACTTGAAGG

*Tbx3* Forward primer: AGGAGCGTGTCTGTCAGGTT

Reverse primer: GCCATTACCTCCCCAATTTT

*Jak2* Forward primer: GACCAGACTCCACTGGCTGT

Reverse primer: GTTCCTGGCAGTGGCTTTAC

*Ptx3* Forward primer: GCTGTGCTGGAGGAACTGC

Reverse primer: AGCTTCATTGGTCTCACAGGA

*Gsn* Forward primer: GCTTTGAGTCGTCCACCTTC

Reverse primer: AAACTGTCCCAGGACACAGG

*Itga2* Forward primer: ACAGACGTGCTCCTGGTAGG

Reverse primer: CTGAACCAAACCGAGCATTT

*Tuba1a* Forward primer: AGCTCATCACAGGCAAGGAG

Reverse primer: AAGAAGCCCTGGAGACCTGT

*Pou5f1* Forward primer: AGAGGGAACCTCCTCTGAGC

Reverse primer: CCAAGGTGATCCTCTTCTGC

*Nanog* Forward primer: CACCCACCCATGCTAGTCTT

Reverse primer: ACCCTCAAACTCCTGGTCCT

*Fgf5* Forward primer: CCTTGCGACCCAGGAGCTTA

Reverse primer: CCGTCTGTGGTTTCTGTTGAGG

*Kdr* Forward primer: TTTGGCAAATACAACCCTTCAGA

Reverse primer: GCAGAAGATACTGTCACCACC

*Hnf4a* Forward primer: ACGCCGGTGAGCATCTGC

Reverse primer: CCGCTAGCTCTGGACAGTGC

*Ring1A* Forward primer: AGCAAAACGTGGGAACTGAG

Reverse primer: GCAGTCCGAGCAGAACCTAT

*Gapdh* Forward primer: ACCACAGTCCATGCCATCAC

Reverse primer: TCCACCACCCTGTTGCTGTA

**Gene expression microarray:data processing**
The open source software R/Bioconductor was used to extract signal presence and intensity using the MAS5 algorithm from GeneChIP Mouse Genome 430 2.0 arrays (Affymetrix, Santa Clara, Calif., USA). Signal intensity of each probe was normalized using a quantile normalization method to compare with other experiments. We calculated the log of the ratio of the intensity in the mutant samples to the intensity in the respective control samples and ignored probes whose signals were not observed significantly in all experiments. The expression change of a gene having multiple probes was calculated using the average of log ratios for all probes assigned to that gene. Enrichment of genes having a GO term was evaluated using hypergeometric distribution of 2x2 contingency table with two conditions, whether a gene is annotated as having the GO term or not, and whether the gene is derepressed (log_2_ ratio of expression change is more than 0.5) or not. GO annotation for each probe (Mouse 430A_2 annotations release 28) was obtained from the vendors website (http://www.affymetrix.com/). GO annotation for the microarray was retrieved from the array vendor’s website. Normalization of microarray signals, calculation of expression changes and statistical evaluation of GO analysis were performed using our in-house programs written in Python.

**ChIP-on-chip data analysis**
ChIP on chip analysis for H3K27me3, Ring1B, 3xFlag-Ring1A, H2AK119u1 and H2A in ESCs was carried out using the Mouse Promoter ChIP-on-chip Microarray Set (G4490A, Agilent, Palo Alto, Calif., USA). Enrichment of immunoprecipitated DNA was calculated and aligned on the TSS. The distance from the TSS was obtained from annotation 014716_D_GeneList_20070207 provided by the vendor. All signals from the array were statistically examined and only signals significantly (*P*<10^-7^) different from background noise were counted. The log ratios of the measured intensities (IP/input: Fold enrichment) were calculated, and a mean of the log ratios in each promoter region (-4 kb to +4 kb around TSS) of a gene was used to represent the index of the gene, since most of polycomb binding and associated histone modifications were observed within ± 4kb of TSSs. Calculation of fold enrichment, normalization between IP and input, and statistical evaluation were performed using our in-house computer program written in C++.

**Three dimensional (3D)-DNA-FISH**

3D-DNA-FISH with spatial preservation of chromatin architecture was performed as described previously . Live or immunostained cells on round coverslips were fixed with 4% PFA for 10 min and washed with cooled PBS twice. Permeabilization was done with 0.5% TritonX-100/PBS for 20 min. After a wash with cooled PBS, coverslips were immersed in 20% glycerol/PBS for 1 h and then subjected to four freeze-thaw cycles with liquid nitrogen. Following a PBS-wash, coverslips washed with PBS were treated with 0.1N HCl for 10 min and 2xSSC for 5min and subsequently kept at 4˚C in 50% ethanol overnight or were used within a week. Coverslips were incubated in 70% formamide/2xSSC (75˚C) for 5 min, cooled 70% ethanol for 5 min, and then 100% ethanol for 5 min. An air-dried coverslip was subjected to hybridization with 20 µl of cocktail (2xSSC/2 mg/ml BSA/10% dextran sulfate) containing denatured mouse Cot-1 DNA (2.5µg) and appropriate volumes of DNA probes that were labeled with Cy3-dCTP (GE Healthcare) and/or Alexa488-dCTP (Molecular Probes) by a Nick Translation kit (Roche). Probe hybridization was carried out for 48-72h at 37˚C and then coverslips were washed with 50% formamide/2xSSC (42˚C) for 5 min twice, 0.1% Tween20/2xSSC (42˚C) for 5 min twice, cooled 70% ethanol for 5 min twice, and 100% ethanol for 5 min once, and finally mounted after drying. FISH signals were detected by our imaging system, which consists of an inverted microscope IX71 with oil immersion objectives (Olympus UPlanSApo 100x NA 1.40 and PlanApo N 60x NA 1.42), a high-speed spinning disc confocal unit (CSU-X1, Yokogawa Electric Corp., Japan) equipped with a CCD camera (ORCA-AR, Hamamatsu Photonics), 488nm (Sapphire CDRHLP, Coherent), and 561nm (85-YCA-025-040, Melles Griot). DNA probes used in these experiments were the 50-kb BAC MMP-4 for *Hoxb1* , RP24-164J9 for *Hoxb13*. FISH signals were detected in the way of 65-nm pixels in X-Y and 300-nm steps in Z. Collected images were constructed by Volocity (PerkinElmer) and then distance between centers of each FISH signal were measured.

**References**

Bolstad, B.M., Irizarry, R.A., Astrand, M., and Speed, T.P. (2003). A comparison of normalization methods for high density oligonucleotide array data based on variance and bias. Bioinformatics *19*, 185-193.

Chambeyron, S., and Bickmore, W.A. (2004). Chromatin decondensation and nuclear reorganization of the HoxB locus upon induction of transcription. Genes Dev *18*, 1119-1130.

Solovei, I., Cavallo, A., Schermelleh, L., Jaunin, F., Scasselati, C., Cmarko, D., Cremer, C., Fakan, S., and Cremer, T. (2002). Spatial preservation of nuclear chromatin architecture during three-dimensional fluorescence in situ hybridization (3D-FISH). Exp Cell Res *276*, 10-23.
